# Motive perception pathways to the release of personal information to healthcare organizations

**DOI:** 10.1186/s12911-022-01986-4

**Published:** 2022-09-13

**Authors:** Michaela Soellner, Joerg Koenigstorfer

**Affiliations:** Chair of Sport and Health Management, Technical University of Munich, Campus D – Uptown Munich, Georg-Brauchle-Ring 60/62, 80992 Munich, Germany

**Keywords:** Artificial intelligence, Attribution, Self-disclosure, Falsification

## Abstract

**Background:**

The goal of the study is to assess the downstream effects of who requests personal information from individuals for artificial intelligence-(AI) based healthcare research purposes—be it a pharmaceutical company (as an example of a for-profit organization) or a university hospital (as an example of a not-for-profit organization)—as well as their boundary conditions on individuals’ likelihood to release personal information about their health. For the latter, the study considers two dimensions: the tendency to self-disclose (which is aimed to be high so that AI applications can reach their full potential) and the tendency to falsify (which is aimed to be low so that AI applications are based on both valid and reliable data).

**Methods:**

Across three experimental studies with Amazon Mechanical Turk workers from the U.S. (n = 204, n = 330, and n = 328, respectively), Covid-19 was used as the healthcare research context.

**Results:**

University hospitals (vs. pharmaceutical companies) score higher on altruism and lower on egoism. Individuals were more willing to disclose data if they perceived that the requesting organization acts based on altruistic motives (i.e., the motives function as gate openers). Individuals were more likely to protect their data by intending to provide false information when they perceived egoistic motives to be the main driver for the organization requesting their data (i.e., the motives function as a privacy protection tool). Two moderators, namely message appeal (Study 2) and message endorser credibility (Study 3) influence the two indirect pathways of the release of personal information.

**Conclusion:**

The findings add to Communication Privacy Management Theory as well as Attribution Theory by suggesting motive-based pathways to the release of correct personal health data. Compared to not-for-profit organizations, for-profit organizations are particularly recommended to match their message appeal with the organizations’ purposes (to provide personal benefit) and to use high-credibility endorsers in order to reduce inherent disadvantages in motive perceptions.

**Supplementary Information:**

The online version contains supplementary material available at 10.1186/s12911-022-01986-4.

## Background

Because of recent advances in technology, the healthcare system produces a vast amount of data. The availability of data types includes behavioral, biological, medical, and environmental data, which are collected through diverse sources (e.g., wearables, medical devices, electronic health records, and social media). Given the availability of these data, it is not surprising that big data has become the main driving force for the transformation of the healthcare industry. The human capability alone to analyze such data reaches its limits. This paves the way for technological assistance. Breakthroughs in algorithmic methods such as machine learning and deep-learning-based artificial intelligence (AI) have helped to unlock the potential of big data for healthcare analytics [1–3].

AI can increase the speed and reduce the costs of high-quality healthcare [4, 5]. Yet the key to creating beneficial AI applications strongly depends on the quality and quantity of relevant health data [6]. The data need to be disclosed and they have to be valid and reliable (if made available). AI applications can create value for patients, clinicians, healthcare organizations, pharmaceutical companies, and health insurers, among others. It is well-known that the entity that requests personal information from individuals influences their likelihood to disclose data, with the highest willingness to disclose data for hospitals [7]. However, the explanatory mechanisms for differences compared to other stakeholders, such as pharmaceutical companies [7], and their boundary conditions often remain unexplored. We argue that there are differences because individuals attribute motives to the requesting entities (particularly for-profit organizations vs. not-for-profit organizations) with different consequences on intentions to disclose. Beside the resulting consequences of who the entities are that request information, we assess when and how entities may increase the likelihood that the request is successful. The latter is particularly important to for-profit organizations such as pharmaceutical companies that can use these data to improve their products and services and innovate [8, 9].

The goal of the present study is to assess the downstream effects of who requests personal information from individuals for AI-based healthcare research purposes—be it a pharmaceutical company (as an example of a for-profit organization) or a university hospital (as an example of a not-for-profit organization)—as well as their boundary conditions on individuals’ likelihoods to release personal information about their health. For the latter, we consider two dimensions: the tendency to self-disclose (which is aimed to be high so that AI applications can reach their full potential) and the tendency to falsify (which is aimed to be low so that AI applications are based on both valid and reliable data). Both dimensions have been shown to be important in past research [10].

We conducted a series of experimental studies and contribute to the literature by (1) introducing motive perception pathways that shape individuals’ likelihoods to disclose personal information depending on the type of requester (for-profit vs. not-for-profit organization) and (2) considering both message appeal and message endorser characteristics as important moderators of the relationship between the requesting entity, motive perception, and likelihood of release of personal information (Additional file 4).

The remainder of this article is organized as follows. We briefly review the relevance of AI in healthcare and introduce our conceptual framework. We then sequentially motivate and present the results of three experimental studies. We conclude with a general discussion of our findings and illustrate the limitations and opportunities for future research.

### Artificial intelligence in healthcare

AI applications in healthcare are expected to advance medical decision-making systems by leveraging the large amounts of patient-level data. Decision-makers such as healthcare organizations or clinicians can benefit from improved workflow and reduced medical errors. Healthcare analytics model risks of adverse events based on clinical and/or non-clinical patterns in data. The prediction of future health-related outcomes, such as medical complications [11], treatment responses [12], patient readmissions [13], and patient mortality [14], increases efficiency and precision to the mutual benefit of patients and healthcare organizations.

AI applications can also consider various patient-specific factors and assist healthcare providers in assessing patients’ risks more granularly and attain the goals of preventive and personalized care [15]. Pattern recognition using deep learning supports clinicians in many disciplines (e.g., radiology, pathology, dermatology, and cardiology); the rapid and accurate interpretation of medical scans can facilitate accurate diagnoses [16]. The tools have also been shown useful in many other clinical settings such as for paramedics in identifying heart attacks or helping anesthesiologists avoid low oxygenation during surgery [17, 18].

Pharmaceutical companies invest in AI since it shows promising results in the realm of drug discovery [6]. Here, the most obvious advantage of algorithms is their capability to increase efficiency by examining millions of molecular structures, searching biomedical literature with high speed as well as designing and making new molecules [8, 9]. Another promising aspect is that they can identify entirely new drugs, operating detached from existing expert techniques [19], and discover previously unidentified drug interactions leveraging pooled datasets [20]. By predicting off-target effects, toxicity, and the right dose for experimental drugs, unintended adverse effects can be reduced [21].

Another benefit of AI is that healthcare can be personalized to individual needs along all stages of care, including prevention, diagnosis, treatment, and follow up [22]. With their value-based care framework, Agarwal et al. (2020) highlight that the availability of data and analytical tools creates an opportunity for healthcare to increase patient empowerment. Information about individuals’ preferences does not only help gain a better understanding of what outcomes really matter to patients, but the information can also improve decision making [23]. Treatment plans can be tailored to individual needs according to their genomic characteristics, personality traits or situational context.

While the amount of health data increases, so do the concerns [24]. Efforts in technological advancement can be diminished when the main source of health data runs dry. Patients may restrict access to their health information if they perceive more risks than benefits. Privacy concerns are a constant topic in healthcare information technology research [7, 25–27]. Since health data are perceived as sensitive, individuals ascribe high risk to revealing such information and are often reluctant to disclose sensitive information [26, 28–30].

Further major concerns are the exposure of personal health information and the legitimate use of health data. One of the main reasons is the fear of real consequences of discrimination in health insurance and employment-based discrimination depending on preexisting health conditions [4]. The growing reluctance of patients to give their data to healthcare organizations is not only related to privacy risks but also to the perception of being exploited. Even if patients release personal information for purposes of AI-based research on improving health, healthcare organizations earn the majority of financial benefits, while the contributors may get nothing (or only little) in return [5]. Since healthcare research is increasingly performed by for-profit companies that serve investors’ needs (according to the rules of the capital market), individuals will be even more cautious with their data. Even though these organizations may protect individuals’ privacy by only using anonymized data, identities can still be leaked by third-party firms that link pieces of data together [31].

Besides their hesitance to self-disclose personal health information, individuals engage in control strategies. In particular, they falsify information—that is, they create and convey wrong information to others [32] to protect their privacy [33, 34]. Misrepresentation facilitates self-protection in response to a request for sensitive information. To reduce their vulnerability to opportunistic behavior, individuals might fabricate such information. This enables them to keep their privacy and simultaneously placate or satisfy others [33]. Misrepresentation of information does not disturb the social exchange, but allows individuals to proceed with an interaction. This behavior is detrimental to the effectiveness of big data technologies in healthcare since it may negatively affect the validity and reliability of results and may thus have further negative downstream consequences. In the healthcare environment, accurate information is critical to achieve high-quality outcomes for patients. To this end, the present research considers both factors of disclosure management: the behavioral intention to self-disclose personal information and the behavioral intention to falsify this information. Information boundary management, which will be explained next, provides the conceptual framework for studying these two behavioral intentions.

### When individuals release true personal information

Communication Privacy Management Theory was initially developed to understand how individuals make decisions regarding the disclosure of information in interpersonal relationships [35, 36]. The theory has also been used to explain individual-organization interactions in both the for-profit and the not-for-profit sector [7, 37]. It uses the metaphor of boundaries to illustrate how individuals control and govern the information flow with others. A boundary represents a psychological contract between the information sender and the receiver and defines the amount, nature, and circumstances of information exchange [38]. When individuals wish to reveal private information, boundaries are opened and the flow of information to and from the self is not restricted, which encourages further information requests. When individuals wish to restrict information exchange, boundaries are closed.

Individuals control their boundaries based on the ratio of benefits and risks associated with the privacy of the information (see the various benefits and risks for AI applications in healthcare above). Important to the present research, Communication Privacy Management Theory has been successfully applied to person-organization relationship contexts [7, 39–41], supporting the relevance of the key variables in the business-to-consumer domain. The formation of boundary rules such as culture, context, and risk-benefit ratio, is determined by criteria that are salient to individuals at the point of time that they make the decision [42]. Another factor that is of particular interest to the present research is the perception of motives [42]. This becomes relevant when individuals wonder why an entity might ask for their personal information. In the following we argue that differences in the type of requester (for-profit vs. not-for-profit organization) will influence individuals’ perception of motives related to the request from the organization with resulting consequences for the release of true personal information.

### The type of requester of personal information and motive perception

Based on differences in objectives, performance criteria, ownership level, and trust [7, 43–51], individuals may attribute different motives to health organizations when these organizations request personal information. This is because individuals make use of cues available in their environment to make causal inferences. While the ownership structure of pharmaceutical companies often reflects the status of for-profit organizations whose activities are governed by capital market-oriented structures, the ownership structure of hospitals often reflects the status of not-for-profit or public organizations, mostly financed by the state, charities or research and education funds.

Attribution Theory illustrates the underlying cognitive process by which individuals assess the motives of others’ behaviors. It is based on the assumption that individuals seek to develop an understanding of the events that they observe or experience [52, 53]. Individuals, exposed to some form of marketing activity of organizations (here: requests for personal healthcare information), make inferences about their motives, which then drive evaluations and behaviors [54–57]. Individuals have been shown to attribute two main types of motives: altruistic motives that aim at the well-being of individuals external to the firm and egoistic motives that focus on the potential benefit to the organization itself. Prior research used various labels for these two motives including socially motivated versus profit-motivated [58] and public-serving versus firm-serving [56].

Altruistic motives are attributed to organizations when individuals perceive that they perform a behavior because they care about others’ welfare [59] and are driven by sincere and benevolent intentions [60]. These attributions affect individuals’ responses positively [61]. Given their not-for-profit ownership (and the mission behind this structure to benefit the community, which might increase trust [7]), we expect individuals to attribute higher altruistic motives to university hospitals’ requests for personal information (as an example of healthcare research-relevant, not-for-profit healthcare organizations) compared to when pharmaceutical companies request personal information (as an example of research-relevant for-profit healthcare organizations) for healthcare research purposes.

Egoistic motives center around ego-driven needs and self-interests of organizations. Goals such as increased market share or publicity are highlighted. Egoistic motives cause negative responses among individuals [58], because their activities are judged as manipulative [60]. Given their for-profit ownership (and the mission behind this structure to benefit the organization), we expect individuals to attribute egoistic motives to pharmaceutical companies as compared to university hospitals, when they request personal information for healthcare research purposes. The former are inferred to exist due to their ability to profit from their relations with consumers [59], while this might not be true for the latter. In this context, consumers might evaluate for-profit organizations from a profit maximization logic, where they expect the organization to act mainly out of self- or egoistic interests [62, 63]. This might not be the case for not-for-profit organizations such as a university hospital. H1a and H1b are stated as follows:

#### Hypothesis 1a

Attributions of altruistic motives for the request of personal information for healthcare research purposes will be lower for pharmaceutical companies compared to university hospitals.

#### Hypothesis 1b

Attributions of egoistic motives for the request of personal information for healthcare research purposes will be higher for pharmaceutical companies compared to university hospitals.

### The downstream relations of perceived motives

The underlying motives that individuals attribute to a health organization’s information request might relate to individuals’ information disclosure tactics. We argue that, first, the perception of altruistic motives will associate with the opening of borders and facilitate information flow between the individual and the healthcare organization (and hence affect self-disclosure of information), and, second, the perception of egoistic motives will prompt individuals to make information-protective behavior more likely (in the form of falsification of information). In what follows, we explain our arguments in more detail.

Individuals are aware that they need to release personal information in exchange for certain benefits to satisfy their needs [64]. The exchange of information is part of what is known as a social contract: individuals have something of value to others and both parties decide to engage in a mutually agreeable trade [65]. Altruistic motives-driven perceptions indicate that healthcare organizations emphasize the creation of social and common benefits. As a consequence, these perceptions might open the boundary and make individuals more likely to disclose personal information. Altruistic motives lower the barrier for action. Hence, altruistic motives should act as a mediator between the type of organization that requests personal information (pharmaceutical company vs. university hospital) and the willingness to self-disclose personal information.

#### Hypothesis 2a

Attributed altruistic motives mediate the relationship between the health care organization that is requesting personal information (pharmaceutical companies vs. university hospitals) and an individual’s self-disclosure intentions.

The social contract between requesters and releasers of personal information comprises commonly understood obligations or social norms for both parties; this is critical for the prevention of opportunistic behaviors [66]. Most importantly to the present study, we can assume that when individuals attribute egoistic motives to the information requester, they might be concerned that the organization may not honor the social contract, so that they act only in their own best interest. The egoistic motive might fuel individuals’ skepticism and lead to negative reactions [56]. To retain control while still reaping the benefits of the exchange, individuals may misrepresent their data [34, 67]. This need for a defensive tactic might stem from the underlying motives that individuals attribute to the information request. Subsequently, individuals will be more likely to misrepresent their data in the information exchange with the health organization. We therefore postulate that egoistic motives act as a mediator between the type of organization that requests personal information (pharmaceutical company vs. university hospital) and the willingness to falsify personal information.

#### Hypothesis 2b

Attributed egoistic motives mediate the relationship between the health care organization that is requesting personal information (pharmaceutical companies vs. university hospitals) and individuals’ falsification intentions

**Fig. 1:**
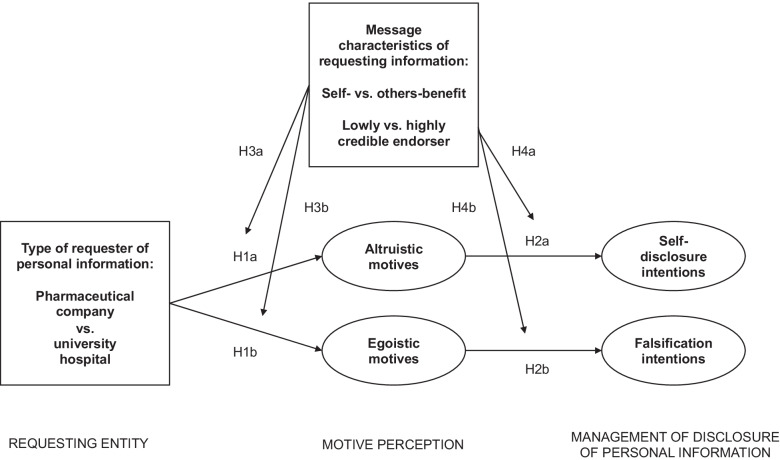
Conceptual model of how and when individuals release personal information for different entities requesting the data. *Notes*. Study 1 tests H1 and H2; Study 2 tests H1-H3; and Study 3 tests H1, H2, and H4

Figure 1 provides an overview of the conceptual model that guided our research. Study 1, which is presented in the following, aims to test H1 and H2.

### Study 1

The purpose of Study 1 is to provide initial evidence that individuals make different motive attributions to not-for-profit (vs. for-profit) healthcare organizations’ requests to share certain personal information with them. Moreover, the study assesses whether attributed altruistic and egoistic motives mediate the relationship between the type of information requester and individuals’ intentions to self-disclose or falsify personal information.

## Method

### Design and sample

We conducted a scenario-based, randomized experiment. The information requester was manipulated between participants, being either a university hospital (as an example of a not-for-profit organization) or a pharmaceutical company (as an example of a not-for-profit organization). Covid-19 was used as the research context for the study (Additional file 1).

We aimed to meet the sample-size recommendations of at least 200 for structural equation models [68], as per our planned analysis. Assuming a small effect size (f^2^ = 0.20; [69]) for the two paths from the experimental manipulation to the two mediators (a paths) and a medium effect size (f^2^ = 0.20; [69]) for the two paths from the mediators to the outcome measures (b paths), as well as a small-to-moderate residual correlation (r = 0.20) between the two mediators and a very small effect size (f^2^ = 0.10) for the direct effect (c’) from the experimental manipulation to the two outcome measures, and specifying a two-tailed value of α = 0.05, the sample size of 200 leads to the projected power of 1 − β = 0.81 (95% CI [0.78, 0.84]) for detecting the two indirect effects—meeting the recommended level of 0.80 [70]. Thus, a sample of at least 200 participants was projected to be adequate to detect differences in perceived motives (and relevant downstream variables) depending on the type of information requester.

A total of 204 respondents recruited from Amazon’s Mechanical Turk participated in Study 1, which was conducted on April 6, 2020. Most of them were male (68.6%). Participants were between 21 and 68 years old with a mean age of 36.2 years and 55.9% had a Bachelor’s degree. Most of the participants either lived in three-person (28.4%) or four-person (29.9%) households. General health was assessed on a five-point scale, ranging from poor (1) to excellent (5). Participants stated that they are in good health (M = 3.82, SD = 0.88). Participants estimated their risk of being infected with the virus on a seven-point rating scale as rather moderate (M = 4.62, SD = 1.72). Also, participants perceived the risk of Covid-19 affecting their health as moderate (M = 4.82, SD = 1.74). Table 1 provides an overview of the sample characteristics.

**Table 1:** Sample characteristics for Study 1, Study 2, and Study 3

Characteristic	Study 1	Study 2	Study 3
Gender (male, %)	68.6	65.2	64.3
Age (18–25 years, %)	11.8	8.5	12.5
(26–35 years, %)	48.5	41.8	45.4
(36–45 years, %)	19.6	19.7	19.8
(46–55 years, %)	11.3	20.9	11.0
(56–65 years, %)	7.4	6.7	10.1
(66 years or more, %)	1.5	2.4	1.2
Education (High school, %)	11.3	6.4	6.1
(Some college, %)	18.1	12.7	24.7
(Bachelor, %)	55.9	48.5	51.2
(Master, %)	12.7	29.4	14.9
(Other, %)	2.0	3.0	3.0
Household size (1, %)	14.7	14.2	13.4
(2, %)	19.1	17.6	21.0
(3, %)	28.4	27.0	32.6
(4, %)	29.9	30.0	25.0
(5 or more, %)	7.8	11.2	7.9
General health (M [SD])	3.82 (0.88)	3.79 (0.92)	3.83 (0.89)
Risk perception of getting infected with Covid-19 (M [SD])	4.62 (1.72)	4.23 (1.75)	4.01 (1.81)
Risk perception of negative health effects of Covid-19 (M [SD])	4.82 (1.74)	4.61 (1.68)	4.29 (1.72)

General health was assessed on a point-point scale (1 = poor, 5 = excellent), risk perceptions were assessed on a seven-point scale (1 = very low, 7 = very high)

### Procedure

After giving consent to participation, participants were asked to picture themselves in a hypothetical scenario. Participants were randomly assigned to one of the two experimental conditions. They either read that given the current situation with the Covid-19 virus, a university hospital announced that they are setting up a comprehensive database of people’s health data for conducting AI-based analyses or they read that given the current situation with the Covid-19 virus, a pharmaceutical company announced that they are setting up a comprehensive database of people’s health data for conducting AI-based analyses. They were also given information about the aim of the database and the organization’s call on the general population to contribute to this database (which was kept identical between the experimental conditions). Participants were subsequently told to access a secure website where they will be asked about which kind of data they are willing to provide.

After reading the scenario, participants completed a questionnaire (Additional file 2). They answered questions about the perceived motives of the healthcare organization for the information request and about their self-disclosure and falsification intentions. The survey ended with several demographics and descriptive variables.

### Variables

Self-disclosure intentions were operationalized as the extent to which individuals were willing to reveal 20 different types of personal information (α = 0.94). The procedure was adopted from previous research [71] and adapted to the context of the study. The items were measured on a seven-point rating scale, with response options ranging from 1 = very unlikely to 7 = very likely.

Falsification intentions were operationalized as the likelihood of individuals to provide false personal information to the information requester (α = 0.93). We measured the construct on a three-item, seven-point rating scale (1 = strongly disagree, 7 = strongly agree), adopted from previous research [72] and adapted to the study’s context (Additional file 1).

Perceived motives were operationalized as a bi-dimensional construct composed of altruistic and egoistic motives. Altruistic attributions reflect an organization’s consideration of the well-being of individuals as underlying motives (α = 0.88), whereas egoistic attributions focused on firm-centered motives (α = 0.85). Both attributed motives were measured on a seven-point rating scale (1 = strongly disagree, 7 = strongly agree). The altruistic motive construct included eight and the egoistic motives included six items [59, 60, 73]. The two- (vs. one-, three- or four-) factorial solution fit the data best.

### Statistical analysis

Means, standard deviations, and bivariate correlations were calculated using SPSS 28.0 (IBM Corp., Armonk, NY, USA) to examine the associations between the variables. A confirmatory factor analysis was done to assess the validity and reliability of the latent variables (Mplus 7.31; [74]). Discriminant validity between the mediators and the outcome variables was given across all three studies, as indicated by the fact that the average variance extracted of each latent variable was larger than the squared correlation with other latent variables [75]. A path model was used to test our hypotheses (Mplus 7.31). The path model included the type of information requester (pharmaceutical company = 1, university hospital = 0), egoistic and altruistic motives as parallel mediators (both based on means constructed with standardized items) and self-disclosure and falsification intentions as the dependent variables (both based on means constructed with standardized items). All direct and indirect paths were included in the model and we report fully standardized measures [76, 77] and the percentage of proportion mediated to indicate the effect size of the mediation model [78].

## Results and discussion

**Fig. 2:**
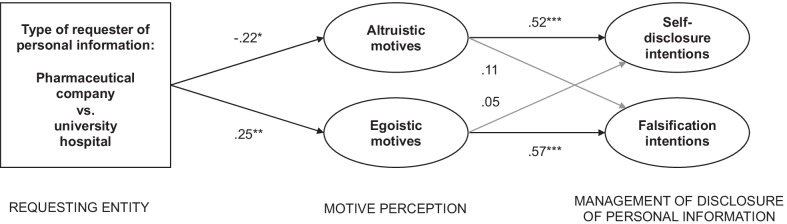
Results of the path analysis on how and when individuals release personal information for different entities requesting the data (Study 1). *Notes*. * p < .10, ** p < .05, *** p < .01. Non-significant paths are shown in grey

The model explained 41.8% of the variance in self-disclosing intentions and 22.5% of the variance in falsification intentions. Figure 2 provides an overview of the results of the path analysis.

The pharmaceutical company was attributed with lower altruistic motives (b = − 0.22, SE = 0.12, *p* = 0.057) and higher egoistic motives (b = 0.25, SE = 0.11, *p* = 0.025) compared to the university hospital, supporting H1a and H1b. Furthermore, the path coefficient between altruistic motives and self-disclosure intentions was positive (b = 0.52, SE = 0.06, *p* < 0.001), providing initial support for H2a. There was no significant relation between egoistic motives and self-disclosure intentions (b = 0.05, SE = 0.05, *p* = 0.35). The path coefficient between egoistic motives and falsification intentions was positive (b = 0.57, SE = 0.07, *p* < 0.001), providing initial support for H2b. There was no significant relation between altruistic motives and falsification intentions (b = 0.11, SE = 0.09, *p* = 0.19).

To test the postulated mediation effects (H2a and H2b), we considered the indirect effects of the type of information requester via egoistic and altruistic motives on the two types of disclosure management. For self-disclosure intentions, there was a significant indirect negative effect of the type of information requester via altruistic motives (b = − 0.12, CI 95% [− 0.024; − 0.004]). There was no significant indirect effect via egoistic motives (b = 0.01, CI 95% [− 0.008; 0.053]). The direct effect of the type of information requester was significant (b = − 0.14, SE = 0.07, *p* = 0.04) and the hypothesized mediation effect accounted for 42.8% of the total treatment effect, which was − 0.25 (CI 95% [− 0.435, − 0.064]). The fully standardized effect-size measure for the indirect effect was − 0.09. The results thus support H2a.

For falsification intentions, egoistic motives (b = 0.14, CI 95% [0.012; 0.271]), but not altruistic motives (b = − 0.03, CI 95% [− 0.123; 0.004]), had a mediating effect. There was no direct effect of the requesting stakeholder on falsification intentions (b = − 0.08, SE = 0.12, *p* = 0.50). The fully standardized effect-size measure for the indirect effect was 0.08. We do not report the percentage that the mediation effect accounted for (compared to the total treatment effect, which was 0.03 (CI 95% [− 0.23, 0.28]), because of the different signs of the direct and indirect effects [77]. The results thus support H2b.

In summary, the results showed that individuals attribute different motives to healthcare organizations when they request personal information. While university hospitals are attributed with the positively connoted motive of altruism, pharmaceutical companies are perceived as egoistically motivated. The results further indicated distinct downstream relations of attributed motives. On the one hand, individuals were more willing to disclose their data if they perceived that the requesting organization acts based on altruistic motives (i.e., the motive functions as gate openers). On the other hand, individuals were more likely to protect their data by intending to providing false information when they perceived egoistic motives to be the main driver for the organization requesting their data (i.e., the motive functions as a privacy protection tool). The research extends previous insights into the consequences of altruistic versus egoistic motives [79, 80] by showing that different disclosure management tactics are associated with the two motives: self-disclosure with altruism and falsification with egoism.

Study 1 focused on the question of how different requesters for personal information make individuals more or less likely to share true information about them and their health. The study did not consider potential moderators that could explain when the two requesters under consideration can profit from (or are harmed by) the motive pathways to self-disclose or falsify personal information. In what follows next, we consider two potential moderators: message appeal (considered in Study 2) and message endorser (considered in Study 3; see Fig. 1).

### Message appeal: self-benefit versus others-benefit

Individuals’ motive attributions can be influenced by communication [59] because message recipients’ causal inferences are strongly linked to features that are present in the environment [52]. Since healthcare organizations attempt to create positive perceptions about their underlying motives, they use various communication tactics [81]. The right tactic is crucial to avoid skepticism and to ensure message persuasiveness [57]. In research into individuals’ pro-social behavior such as donations or ethical consumption, it was found that whether the message highlights the benefits for others or the benefits for the individual affects their approach behavior [82]. In the following, we provide arguments for why such message appeals matter in the context of healthcare providers asking for personal information.

Others-benefit appeals highlight other individuals or the society at large as the main beneficiaries [83], while self-benefit appeals highlight the giver as the main beneficiary of the exchange [82]. We argue that individuals will respond differently depending on who communicates others- (vs. self-) benefits as regards the intentions to disclose personal information. Others-benefits appeals are especially effective in non-commercial exchanges [84] and in the context of social goods, because they trigger an empathy-helping response in individuals [83]. Not-for-profit healthcare organizations such as university hospitals put their efforts into providing benefits for community and the society at large. Hence, others-benefit appeals might be more persuasive in influencing altruistic motives than self-benefit appeals for university hospitals’ (vs. pharmaceutical companies’) request of personal information. Given university hospitals’ not-for-profit status, the message appeal is in harmony with individuals’ attributed motives. This should strengthen individuals' attributions of altruistic motives and might thus have a positive indirect effect on individuals’ self-disclosure intentions of personal information.

#### Hypothesis 3a

The message appeal moderates the relationship between the type of information requester and self-disclosure intentions via altruistic motives, such that the indirect effect will be stronger for the others-benefit (vs. self-benefit) message appeal.

Egoistic motive attributions play an important role for the association with falsification intentions, particularly when pharmaceutical companies request personal information. They are at least partly governed by the rules of the capital market. Even though they operate to increase overall societal well-being, they simultaneously need to maintain attractiveness to owners and potential investors. We expect that differences in message appeal (others- vs. self-benefit) influence the relationship between the type of requester of personal information and the intentions to disclose true information via egoistic motive perception. Based on Forehand and Grier's [56] work, who showed that egoistic attributions only lowered firm evaluations when they were inconsistent with the firm’s publicly expressed motive, one can assume that a for-profit healthcare organization that sends an others-benefit message might cause suspicion about the underlying intent of the message. The argument is supported by findings from Becker-Olsen et al. [58], who found that the fit between what the company stands for and what communication content it uses is important. The persuasiveness of the message is likely to be diminished and individuals are more likely to infer ulterior motives when there is a lack of fit (here: between company goals and communication appeals). We therefore assume that the indirect effect on falsification intentions will be higher for others- (vs. self-) benefit message appeals when pharmaceutical companies (vs. university hospitals) request personal information.

#### Hypothesis 3b

The message appeal moderates the relationship between the type of information requester and falsification intentions via egoistic motives, such that the indirect effect will be stronger for the others-benefit (vs. self-benefit) message appeal.

### Study 2

The purpose of Study 2 is to assess whether message appeal—the focus on others-benefits versus self-benefits in communication campaigns about the release of personal information—influences the two pathways of how individuals self-disclose or falsify information depending on who requests the data. We expect that who the requester is matters more when other-benefits (vs. self-benefits) are highlighted.

## Method

### Design and sample

A 2 (information requester: pharmaceutical company vs. university hospital) × 2 (message appeal: self-benefit vs. other-benefit) design was used, manipulating both factors between participants. The study was conducted on May 5, 2020, and we used the same scenario as in Study 1.

As in Study 1, we aimed to meet the sample-size recommendations of at least 200 for structural equation models [68]. Based on the interpretation of bootstrapping results of simulation studies for moderated mediation models, a sample size between 200 and 500 has been suggested for the criteria specified for the purpose of the present study (see Study 1: two-tailed value of α = 0.05, power of 1 − β = 0.80, small effect sizes) [85]. A total of 330 participants from the U.S. (M_Age_: 38.8 years, 65.2% males) were recruited from Amazon’s Mechanical Turk and they were randomly assigned to one of four experimental groups. Because of the large impact of the pandemic in the U.S. in May 2020, we excluded participants who stated that they or somebody from their family had been infected. Participants stated that they are in good health (M = 3.79, SD = 0.92). The estimated risk of being infected with Covid-19 (M = 4.23, SD = 1.75) and the perceived risk of Covid-19 affecting health (M = 4.61, SD = 1.68) were perceived as moderate.

### Procedure

Participants read that a university hospital (pharmaceutical company) announced that they are setting up a comprehensive database of people’s health data. After the healthcare organization’s call on the general population to contribute to this database, participants read an announcement with one of the two different message appeals (Additional files 1 and 3). Participants subsequently followed the same procedure as in Study 1.

### Variables

Study 2 used the same scales as in Study 1 to assess individuals’ self-disclosure (α = 0.96) and falsification intentions (α = 0.94) as well as attributions of egoistic (α = 0.86) and altruistic motives (α = 0.93).

### Statistical analysis

The software used for the statistical analysis of the data was identical to Study 1. Following Stride et al.'s [86] guidelines, a path model was created with two dependent variables, two parallel mediators, and one moderator. As in Study 1, the information requester was included as independent variable (pharmaceutical company = 1, university hospital = 0). The model tests for moderating effects of message appeal (self-benefit = 1, others-benefit = 0) on both independent variable-mediator paths (a paths) and mediator-dependent variable paths (b paths). Attributed egoistic and altruistic motives were modeled as mediators on self-disclosure and falsification intentions as dependent variables. We report fully standardized measures to indicate the effect size of the mediation model [76, 77].

## Results and discussion

The model explained 48.5% of the variance in self-disclosing intentions and 12.5% of the variance in falsification intentions (Table 2).

**Table 2:** Results of the moderation effect of message appeal on the relationship between the type of information provider on intentions to manage disclosure of personal information via motive perception (Study 2)

Direct effects on motive perception	b	SE	*p*
Requester → Egoistic motives	.43	.12	< .001
Message appeal → Egoistic motives	.17	.13	.18
Requester × Message appeal → Egoistic motives	− .38	.16	.02
Requester → Altruistic motives	− .42	.14	.002
Message appeal → Altruistic motives	.13	.10	.18
Requester × Message appeal → Altruistic motives	− .02	.18	.91

Direct effects on self-disclosure and falsification	b	SE	*p*
Requester → Self-disclosure	− .07	.06	.28
Altruistic motives → Self-disclosure	.61	.06	< .001
Egoistic motives → Self-disclosure	.07	.06	.26
Message appeal → Self-disclosure	− .13	.06	.02
Altruistic motives × Message appeal → Self-disclosure	.06	.08	.47
Egoistic motives × Message appeal → Self-disclosure	− .02	.08	.83
Requester → Falsification	− .20	.10	.05
Altruistic motives → Falsification	− .07	.09	.42
Egoistic motives → Falsification	.43	.08	< .001
Message appeal → Falsification	.01	.10	.95
Altruistic motives × Message appeal → Falsification	.02	.13	.86
Egoistic motives × Message appeal → Falsification	.03	.12	.79

Moderation effects of message appeal	b	CI 95%	Effect size
*Conditional indirect effect via altruistic motives on self-disclosure*			
Self-benefit	− .29	[− .475; − .133]	− .19
Others-benefit	− .26	[− .415; − .088]	− .17
*Conditional indirect effect via egoistic motives on self-disclosure*			
Self-benefit	.00	[− .006; .035]	.00
Others-benefit	.03	[− .020; .097]	.02
*Conditional indirect effect via altruistic motives on falsification*			
Self-benefit	.02	[− .074; .106]	.01
Others-benefit	.03	[− .036; .127]	.02
*Conditional indirect effect via egoistic motives on falsification*			
Self-benefit	.03	[− .073; .129]	.02
Others-benefit	.19	[.076; .331]	.10

b, Unstandardized path coefficient; SE, Standard error; *p*, Significance; CI 95%, 95% Confidence interval. Self-disclosure, Intentions to self-disclose personal information; Falsification, Intentions to falsify personal information. Effect sizes are fully standardized.

In support of H1a and H1b, the pharmaceutical company was attributed with lower altruistic motives (b = − 0.42, SE = 0.14, *p* = 0.002) and higher egoistic motives (b = 0.43, SE = 0.12, *p* < 0.001) compared to the university hospital. The message appeal had no direct effect on attributed motives (b_Egoistic_ = 0.17, SE = 0.13, *p* = 0.18, b_Altruistic_ = 0.13, SE = 0.10, *p* = 0.18). While the interaction effect of the type of requester and message appeal (a path) was significant on egoistic motives (b = − 0.38, SE = 0.16, *p* = 0.02), it was non-significant for altruistic motives (b = − 0.02, SE = 0.18, *p* = 0.91). In support of H2a and H2b, the path coefficients (b paths) between altruistic motives and self-disclosure (b = 0.61, SE = 0.06, *p* < 0.001) and between egoistic motives and falsification were significant (b = 0.43, SE = 0.08, *p* < 0.001).

To test H3a and H3b, conditional indirect effects were considered via bootstrapping. The indirect effects of the type of requester on self-disclosure intentions via altruistic motives were negative and significant for both message appeals (b_Others-benefit_ = − 0.26, CI 95% [− 0.415; − 0.088] and b_Self-benefit_ = − 0.29, CI 95% [− 0.475; − 0.133], respectively). The index of moderated mediation, which tests for differences in the two indirect paths, was 0.04 with a bootstrap 95% confidence interval of [− 0.174; 0.180], indicating that pharmaceutical companies were less likely to successfully request data from individuals via shaping altruistic motive perception for both the others-benefit and the self-benefit message. The results do not support H3a. The hypothesized mediation effect accounted for 79.6% and 81.4%, respectively, of the total treatment effect on self-disclosure intentions via altruistic motives (in the others-benefit and self-benefit conditions, respectively), which were − 0.32 (CI 95% [− 0.520, − 0.095]) and − 0.36 (CI 95% [− 0.554, − 0.169]), respectively.

The indirect effects of the type of requester on falsification intentions via egoistic motives was non-significant in the self-benefit condition (b = 0.03, CI 95% [− 0.073; 0.129]) but positive and significant in the others-benefit condition (b = 0.19, CI 95% [0.076; 0.331]). The index of moderated mediation was 0.16 with a bootstrap 95% confidence interval of [0.005; 0.326], indicating that pharmaceutical companies were more likely to request falsified data from individuals via shaping egoistic motive perception in the others-benefit (but not in the self-benefit) message appeal condition. The results support H3b. The finding is interesting and reveals the challenges that for-profit healthcare organizations are faced with: given their status, the communication of others-benefits might not match with individuals’ expectations and this likely led to the negative outcomes [56]; here: likelihood to falsify personal information to protect one’s privacy but to still be able to interact with the provider). We note that, for falsification intentions, we do not report the percentage that the mediation effect via egoistic motives accounted for (compared to the total treatment effect, which were − 0.02 (CI 95% [− 0.239, 0.199]) and − 0.18 (CI 95% [− 0.388, 0.040], in the others-benefit and self-benefit conditions, respectively) because of the different signs of the direct and indirect effects.

In summary, the results replicate the findings from Study 1 with regard to H1 and H2. In addition, the study showed that others-benefit message appeals increased the attributions of egoistic motives for pharmaceutical companies (vs. university hospitals), which resulted in higher falsification intentions. There was no differential effect on self-disclosure intentions for the two message appeals. This might be due to the fact that individuals tended to be not only community-oriented but also selfish; self-benefits appeals can be effective too, because the behavior serves their own needs [83].

One limitation of Study 2 is that it considered the message content without reference to a potential message endorser. During the Covid-19 pandemic and for health promotion and disease prevention purposes in general, actors such as politicians or public health representatives often endorse messages directed at individuals [87, 88]. Such endorsers were found to influence people’s pro-social behaviors [89] and might, in the context of the present research, influence how and when intentions to disclose personal information are formed. In the following, we detail how a message endorser might influence individuals’ intentions to self-disclose or falsify personal information.

### Message endorser characteristics: highly versus less credible endorsers

An external source from the government with decision-making power in public health might influence whether and when individuals share true personal information with a health organization for research purposes. In marketing, the effectiveness of celebrity endorsements for corporate communication activities has been evidenced across various studies [90], and previous research has identified source credibility as a key construct for persuasion [62, 91, 92]. Source credibility has two components: expertise and trustworthiness [93]. Expertise reflects the extent to which a source is perceived as being knowledgeable about a topic; trustworthiness refers to the honesty of the source [91]. Messages delivered or endorsed by a credible source were found to be more readily accepted than messages delivered or endorsed by a less credible sources [94, 95].

Applied to the context of the present study, a highly (versus less) credible endorser who emphasizes the importance of data collection can be assumed to strengthen the positive effect of attributed altruistic motives on individuals' self-disclosure intentions. Since individuals respond more positively when altruistic motives are paired with high-credibility endorsers [96], we can assume that the indirect effect of the type of information requester on intentions to self-disclose personal information via altruistic motives will be affected by whether a highly (versus less) credible public person endorses the message for the request.

#### Hypothesis 4a

The message endorser moderates the relationship between the type of information requester and individuals’ self-disclosure intentions via altruistic motives, such that the indirect effect will be stronger when a highly (vs. less) credible public person endorses the requesting health organization’s efforts in data collection.

In addition to the facilitating role in influencing self-disclosure intentions, the credibility of an endorser might also influence the relationship between egoistic motives and intentions to falsify personal information. Individuals may contest the source of a message when individuals dismiss the credibility of the endorser [97]. This response to reduce or counter persuasion attempts is known as source derogation. Consequently, if a less credible endorser with decision-making power in the government emphasizes a health organization’s efforts of data collection, the relationship between egoistic motives and falsification intentions might be stronger than for a highly credible endorser, particularly when a for-profit (vs. not-for-profit) organization requests the data.

#### Hypothesis 4b

The message endorser moderates the relationship between the type of information requester and individuals’ falsification intentions via egoistic motives, such that the indirect effect will be stronger when a less (vs. highly) credible public person endorses the requesting health organization’s efforts in data collection.

### Study 3

The purpose of Study 3 is to assess whether a message endorser’s credibility in communication campaigns about the release of personal information influences the two pathways of how individuals self-disclose or falsify information depending on who requests the data. We expect that a highly credible source strengthens the indirect pathway on intentions to self-disclose personal information, whereas it weakens the indirect pathway to falsify personal information.

## Method

### Design and sample

To test our hypotheses, we used a 2 (information requester: pharmaceutical company vs. university hospital) × 2 (message endorser’s credibility: high vs. low) design, manipulating both factors between participants.

The study took place on May 5, 2020 and participants were only allowed to participate if they had not taken part in Study 2. The same procedure was applied as for Study 2 and we aimed for a similar sample size. A total of 328 Amazon’s Mechanical Turk participants from the U.S. (M_Age_: 36.7 years, 64.3% males) were randomly assigned to one of four experimental groups. Participants stated that they are in good health (M = 3.83, SD = 0.89). Perceived risks of getting infected with the virus (M = 4.01, SD = 1.81) and perceived risks of Covid-19 affecting individuals’ health were moderate (M = 4.29, SD = 1.72).

### Procedure

Covid-19 provided the study context. Participants read that a university hospital (pharmaceutical company) announced that they are setting up a comprehensive database of people’s health data. Following this, participants read that a public person (either M.D. Deborah Birx or then-U.S. President Donald Trump) encourages people to provide personal health data to healthcare organizations to support them in their efforts. The public person furthermore highlighted the importance of up-to-date and real health data in a press conference for experts in health and medicine. The study continued and ended as described before (Additional files 1 and 4).

We selected Deborah Birx as the public person with a presumably high credibility. She is a renowned health official who is responsible for government responses to Covid-19. Donald Trump, on the other hand also worked for the government, but was found to provide misleading statements about various healthcare topics even before the Covid-19 pandemic [98], potentially leading to low-credibility perceptions. More specifically, he has been criticized for the slow response to Covid-19 [99], making the U.S. the country with the highest number of Covid-19 disease-related deaths at the time when the study was conducted [100].

### Variables

Study 3 used the same scales as the previous studies to assess individuals’ self-disclosure (α = 0.96) and falsification intentions (α = 0.94) as well as attributions of egoistic (α = 0.85) and altruistic motives (α = 0.94). To test whether our assumptions about the selected endorsers were correct or not, we assessed source credibility (α = 0.98) with a 14-item, seven-point rating scale adopted from Ohanian [92] and Reysen [101]. We also measured perceived domain-specific expertise of the endorser with regard to Covid-19. To do so, we adopted five items from Ohanian [92] and adapted them to the study context (α = 0.97).

### Statistical analysis

The software used for the statistical analysis of the data was identical to Study 2. We specified a similar model compared to Study 2, replacing message appeal with message endorser’s credibility (low = 1, high = 0).

## Results

The assumption check about the credibility of the two selected endorsers showed that both credibility (M_Birx_ = 5.14 and M_Trump_ = 3.97, F(1,326) = 84.37, *p* < 0.001) and domain-specific expertise with regard to Covid-19 (M_Birx_ = 5.47 and M_Trump_ = 3.77, F(1,326) = 145.15, *p* < 0.001) differed between the two message endorsers in the expected direction.

The model explained 52.6% of variance in self-disclosure intentions and 18.0% of variance in falsification intentions. Table 3 provides an overview of the results of the moderated mediation analysis.

**Table 3:** Results of the moderation effect of message endorser on the relationship between the type of information provider on intentions to manage disclosure of personal information via motive perception (Study 3)

Direct effects on motive perception	b	SE	*p*
Requester → Altruistic motive	− .27	.14	< .05
Endorser → Altruistic motive	− .04	.12	.77
Requester × Endorser → Altruistic motive	.11	.18	.55
Requester → Egoistic motive	.23	.12	.06
Endorser → Egoistic motive	− .05	.13	.71
Requester × Endorser → Egoistic motive	.05	.17	.76

Direct effects on self-disclosure and falsification	b	SE	*p*
Requester → Self-disclosure	.03	.06	.68
Altruistic motive → Self-disclosure	.69	.05	< .001
Egoistic motive → Self-disclosure	− .01	.06	.93
Endorser → Self-disclosure	− .06	.06	.35
Altruistic motive × Endorser → Self-disclosure	− .09	.07	.19
Egoistic motive × Endorser → Self-disclosure	.04	.09	.63
Requester → Falsification	− .16	.10	.12
Altruistic motive → Falsification	.16	.08	.05
Egoistic motive → Falsification	.47	.08	< .001
Endorser → Falsification	.14	.09	.12
Altruistic motive × Endorser → Falsification	− .11	.12	.38
Egoistic motive × Endorser → Falsification	.20	.11	.07

Moderation effects of message endorser	b	CI 95%	Effect size
*Conditional indirect effect via altruistic motive on self-disclosure*			
Low credibility	− .10	[− .250; .043]	− .07
High credibility	− .18	[− .387; − .016]	− .12
*Conditional indirect effect via egoistic motive on self-disclosure*			
Low credibility	.01	[− .022; .066]	.01
High credibility	− .00	[− .039; .025]	− .00
*Conditional indirect effect via altruistic motive on falsification*			
Low credibility	− .01	[− .071; .013]	.01
High credibility	− .04	[− .144; − .001]	.02
*Conditional indirect effect via egoistic motive on falsification*			
Low credibility	.19	[.043; .362]	.10
High credibility	.11	[− .001; .244]	.06

See Table 2 for abbreviations

The pharmaceutical company was attributed with lower altruistic motives (b = − 0.27, SE = 0.14, *p* < 0.05) compared to the university hospital. There was a marginally significant effect on egoistic motives (b = 0.23, SE = 0.12, *p* = 0.06). The results thus largely support H1. The path coefficients between altruistic motives and self-disclosure intentions (b = 0.69, SE = 0.05, *p* < 0.001) and between egoistic motives and falsification intentions were positive (b = 0.47, SE = 0.08, *p* < 0.001), supporting H2.

The interaction effect of the message endorsers’ credibility and egoistic motives on falsification intention was marginally significant (b = 0.20, SE = 0.11, *p* = 0.07), while there was no significant interaction effect of message endorsers’ credibility and altruistic motives on self-disclosure intentions (b = − 0.09, SE = 0.07, *p* = 0.19). When a pharmaceutical company (vs. university hospital) acts as the information requester, the analysis revealed a negative and significant conditional indirect effect in situations of high credibility of the endorser on self-disclosure intentions via altruistic motives (b = − 0.18, CI 95% [− 0.387; − 0.016]). There was no such evidence for the low-credibility message endorser (b = − 0.10, CI 95% [− 0.250; 0.043]). Even though these results indicate that university hospitals were more likely to successfully request data from individuals in the high-credibility (but not in the low-credibility) message endorser condition by shaping altruistic motive perception compared to pharmaceutical companies, the index of moderated mediation (− 0.09) was non-significant (bootstrap 95% confidence interval of [− 0.323; 0.150]). The results therefore only partly support H4a. We do not report the percentage that the mediation effect via egoistic motives accounted for (compared to the total treatment effects, which were − 0.16 (CI 95% [− 0.391, 0.058]) and − 0.07 (CI 95% [− 0.207, 0.104]) in the high and low endorser credibility conditions, respectively) because of the different signs of the direct and indirect effects.

The assessment of the conditional indirect effects of the highly credible endorser on falsification intentions via egoistic motives revealed a marginally significant positive effect (b = 0.11, CI 95% [− 0.001; 0.244]), which increased in magnitude for the low-credibility endorser (b = 0.19, CI 95% [0.043; 0.362]). The index of moderated mediation was − 0.08 with a bootstrap 95% confidence interval of [− 0.288; 0.132]. Thus, even though the results indicate that pharmaceutical companies were more likely to receive falsified data from individuals in the low-credibility (but less so in the high-credibility) message endorser condition by shaping egoistic motive perception, the results only partly support H4b. We do not report the percentage that the mediation effect via egoistic motives accounted for (compared to the total treatment effect, which were − 0.05 (CI 95% [− 0.267, 0.179]) and 0.04 (CI 95% [− 0.220, 0.275]), in the high and low endorser credibility conditions, respectively) because of the different signs of the direct and indirect effects.

The results of Study 3 showed that the credibility of an external message endorser influences how individuals respond to requests form healthcare research entities to disclose true personal information. The study thus identified another moderator of the relationship between the type of information requester and self-disclosure intentions of true personal data (beside message appeal, considered in Study 2). While message appeal interacts with the type of requester and exerts its influence on motive perception, message endorser credibility interacts with the motive perception and exerts its influence on the two outcome variables. For the latter, the difference test for indirect effects was non-significant.

## General discussion

### Theoretical and practical implications

The purpose of the study was to find out how individuals manage the release of sensitive personal information depending on who requests the data that will be used for healthcare research purposes. We introduce motive perception pathways that shape individuals’ likelihood to disclose personal information depending on the type of requester (for-profit vs. not-for-profit organization). Also, we identify message appeal and message endorser credibility as important moderators of the relationship between the requesting entity, motive perception and likelihood of disclosure of personal information. In what follows, we discuss the contribution of our study in more detail.

First, we have argued, and provided empirical evidence, that it is important to incorporate both self-disclosure and falsification intentions to fully understand individuals’ information management. While prior studies in the healthcare context primarily investigated disclosure and its complement non-disclosure [7, 26, 102–104], we believe that this does not fully reflect how individuals handle information flows. Such an approach specifies only the amount of data that individuals are willing or not willing to share, but disregards the aspect of its accuracy. Other studies addressed this aspect by investigating individuals’ misrepresentation intention as a tool to protect personal information [34, 64, 72, 105]. While both streams of research identified important factors that facilitate or inhibit information sharing, a combined view on individuals’ information management is partly lacking. It is essential to determine what prompts individuals to share personal information truthfully and what causes misrepresentation intentions. To address this, we applied a dual approach in this study by incorporating self-disclosure as well as falsification. Thus, we advance the understanding of how individuals govern their personal health information and show that information management goes beyond pure sharing or non-sharing of data.

Second, we found that individuals were more likely to attribute egoistic motives to for-profit organizations than to non-profit healthcare organizations, with differential downstream relations. To our knowledge, this is the first study that investigated motive attributions in the healthcare setting and information management context. Prior studies mainly investigated individuals’ attributed motives for branding purposes, such as for the context of cause-related marketing [80], corporate social responsibility [57, 58, 73] and sponsorship [59, 79]. In the context of our study, the two attributed motives—altruistic and egoistic motives—explain why individuals open or close information boundaries in response to an information request. Boundaries open up when individuals attribute altruistic motives to information requests. The response to the information request is not restricted but opens the gate for the flow of information between the parties. By contrast, if individuals perceive egoistic motives driving the information request, they are more likely to provide inaccurate information. Egoistic motives increase the perceived risk of opportunistic behaviors, which increase individuals’ vulnerability. Consequently, individuals apply rules to protect themselves and maintain their privacy [72].

Third, the results of Study 2 and 3 provide evidence that the communication content and endorser affect individuals’ management of personal information. The results from Study 2 complement prior findings that found individuals to respond more positively to self-benefit appeals [106, 107]. We have argued that an others-benefit message sent by a for-profit organization would be likely to amplify perceptions of opportunistic behaviors. The altruistic appeal might be counter-intuitive to the purpose of the organization and might raise concerns about persuasion attempts. These concerns shift the focus on egoistic motives, which increases the likelihood of flawed data. Furthermore, in extension of prior research on persuasion, which highlights the positive effects of highly credible message endorsers in commercial settings only [59, 91, 108], the results from Study 3 provide a more nuanced view, considering for-profit and not-for-profit organizations. A credible endorser can particularly help not-for-profit (vs. for-profit) organizations persuade individuals to open their information boundaries and reveal their health data truthfully. For-profit (vs. not-for-profit) organizations that are supported by a lowly credible endorser, however, increase the likelihood that information gates are closed and that data are misrepresented.

This research also provides practical implications for healthcare organizations. In particular, for-profit organizations should frame persuasion messages for the request of personal data carefully to avoid negative reactions on individuals’ intentions to release true information about them. They might work together with high-credibility endorsers, and they might focus on matched interests with providers of personal information (i.e., the benefit for the individual). Since experts with a medical background are often perceived as more credible than officials from government agencies in providing health information [109], particularly when they did not receive educational degrees in medicine, they might take into account domain-specific expertise and select endorsers accordingly.

### Limitations and future research

The study is not without limitations. First, all three studies were done in the healthcare context with particular consideration of Covid-19. Since the pandemic has affected stakeholders of various kinds, this might have influenced individuals’ intentions to disclose personal information depending on the requester. Covid-19 is an infectious disease with important public health implications of infection. That is, there are consequences of the disease for other people in ways that are not the case for non-communicable diseases. Therefore, the results cannot be generalized across diseases. To identify potential peculiarities of the Covid-19 context, future research might replicate the results for other infectious and non-communicable diseases (e.g. cancer and rare diseases) or even outside the context of healthcare.

Second, the sample that was considered in the studies is not representative of the general population in the U.S. Although Amazon Mechanical Turk workers have been reported to consistently report their demographic and personality characteristics across studies [110] and are considered appropriate to be recruited for theory-based hypothesis testing purposes [111, 112], as done in our study, these workers may have been driven by different levels of extrinsic and intrinsic personal motives to complete the survey [113]. The levels of extrinsic and intrinsic motive perception may have affected how they perceived motives for obtaining personal health data from others (in our case, information requesters).

Third, both self-disclosure and falsification of personal data referred to behavioral intentions in the present study. While the scales used have been shown to be valid and reliable [67, 68], the assessment of actual self-disclosure or actual falsification of personal data might be more informative in the sense that social desirability can be reduced or ruled out [114].

Fourth, the model that we tested can be criticized for the omission of variables [115]. For example, it has been argued that emotions [7] or benefit–cost assessments [116] matter in explaining individuals’ management of personal (health) information. Indeed, full evidence that no omitted variables are at play in the assessment of the mediator-outcome correlation is impossible to provide [77]. Yet, in the present study, we measured mediators and outcome variables with different instruments and item types—tools to reduce omitted variable bias [77]. Future research may extend the model, and compare their explanatory power compared to other models, to assess the importance of a broader range of variables and identify suitable theoretical bases. Furthermore, while our research focused on message framing and message endorser credibility, other factors such as two-sided communication [97] might influence how and when individuals disclose personal information. Future research might find out whether two-sided messages are particularly helpful to for-profit organizations to increase the perception of altruism and decrease the perception of egoism to make individuals share true personal data with them.

Lastly, individuals’ attributions might go beyond the perception of altruistic versus egoistic motives. There is still no consensus on the classification of attributions. Other studies have considered more than two dimensions (e.g. strategic-, stakeholder-, and value-driven), which might also be applicable to healthcare contexts [57, 60, 73, 79].

## Conclusions

AI might help unlock the potential of big data for healthcare analytics. Yet, personal information about individuals is needed to get large datasets that feed AI tools. The bottleneck for the availability of these data to healthcare research often centers around individuals’ consent for their data to be used as well as the validity of the patient-reported data. The present research shows that individuals are more willing to disclose data if they perceive that the requesting organization acts based on altruistic motives (i.e., the motives function as gate openers, as shown for university hospitals). Individuals are more likely to protect their data by intending to provide false information when they perceived egoistic motives to be the main driver for the organization requesting their data (i.e., the motives function as a privacy protection tool, as shown for pharmaceutical companies). The findings on the boundary conditions of these effects might be helpful to obtain valid and reliable data from individuals to support AI solutions in healthcare.

## Supplementary Information

**Additional file 1**. Electronic Appendix.

**Additional file 2**. Questionnaire for Study 1.

**Additional file 3**. Questionnaire for Study 2.

**Additional file 4**. Questionnaire for Study 3.

## Data Availability

Datasets used and analyzed during this current study are available from the corresponding author upon reasonable request.

## Abbreviation

AI: Artificial intelligence
