# Supplementary material for: Motive perception pathways to the release of personal information to healthcare organizations
Source: BMC Med Inform Decis Mak. 2022 Sep 13;22:240. doi: 10.1186/s12911-022-01986-4 (PMC9468521; doi:10.1186/s12911-022-01986-4)
Supplement: Supplementary file 1 — Additional file 1. Electronic Appendix. [file 12911_2022_1986_MOESM1_ESM.docx]

**Electronic Appendix**

**Appendix A. Variables for Management of Personal Information (Studies 1-3)**

| **Items** | **Study 1** | | | | **Study 2** | | | | **Study 3** | | | |
| --- | --- | --- | --- | --- | --- | --- | --- | --- | --- | --- | --- | --- |
|  | **PC** | | **UH** | | **PC** | | **UH** | | **PC** | | **UH** | |
| **Self-disclosure intentions.** Please specify the extent to which you would be willing to reveal the following personal information**:** | **M** | **SD** | **M** | **SD** | **M** | **SD** | **M** | **SD** | **M** | **SD** | **M** | **SD** |
| **Demographics**  e.g. age, gender | 5.60 | 1.48 | 5.96 | 1.32 | 5.31 | 1.70 | 5.87 | 1.20 | 5.38 | 1.72 | 5.71 | 1.52 |
| **Current health condition**  e.g. symptoms, illnesses, chronic disease, hospitalization | 5.46 | 1.63 | 5.77 | 1.28 | 4.85 | 1.91 | 5.48 | 1.45 | 4.88 | 2.00 | 5.27 | 1.63 |
| **General health**  e.g. smoking habits, BMI, physical activity, diet, weight, sleeping habits, alcohol consumption | 5.39 | 1.52 | 5.76 | 1.35 | 4.83 | 1.85 | 5.51 | 1.58 | 4.91 | 1.93 | 5.26 | 1.66 |
| **Surgery**  e.g. transplants, plastic surgery, hip replacement | 5.09 | 1.69 | 5.70 | 1.30 | 4.59 | 1.98 | 5.30 | 1.79 | 4.79 | 1.90 | 5.17 | 1.75 |
| **Medication**  e.g. prescribed mediation, over the counter medication, medication adherence | 5.11 | 1.70 | 5.66 | 1.30 | 4.67 | 2.03 | 5.35 | 1.61 | 4.80 | 1.95 | 5.01 | 1.75 |
| **Occupation** | 5.11 | 1.83 | 5.64 | 1.41 | 5.06 | 1.84 | 5.46 | 1.61 | 4.88 | 1.95 | 5.17 | 1.76 |
| **Lifestyle**  e.g. amount of leisure time, working hours, mobility/transportation behavior | 5.03 | 1.65 | 5.49 | 1.38 | 4.84 | 1.91 | 5.31 | 1.66 | 4.79 | 1.91 | 4.98 | 1.75 |
| **Medical history**  e.g. previous illnesses, injuries, surgeries | 4.96 | 1.63 | 5.51 | 1.44 | 4.52 | 2.04 | 5.44 | 1.52 | 4.77 | 1.96 | 5.07 | 1.76 |
| **Test results**  e.g. blood pressure, cholesterol level, screening results, mammogram results | 4.92 | 1.86 | 5.46 | 1.44 | 4.61 | 2.01 | 5.35 | 1.60 | 4.76 | 1.87 | 5.07 | 1.72 |
| **Substance abuse**  e.g. drug, medication or alcohol abuse | 4.94 | 1.84 | 5.42 | 1.42 | 4.31 | 1.98 | 5.04 | 1.87 | 4.46 | 2.11 | 4.59 | 1.96 |
| **Emotional information**  e.g. happiness, sadness, fear | 4.92 | 1.78 | 5.20 | 1.56 | 4.53 | 1.89 | 5.11 | 1.76 | 4.62 | 2.11 | 4.71 | 1.87 |
| **Sexual health information**  e.g. sexual transmitted diseases, HIV | 4.79 | 1.84 | 5.26 | 1.65 | 4.40 | 2.09 | 5.01 | 1.92 | 4.50 | 2.15 | 4.72 | 1.94 |
| **Mental health information**  e.g. psychological therapy or counseling, depression, anxiety disorder, suicide attempts, eating disorder | 4.97 | 1.79 | 5.00 | 1.84 | 4.12 | 1.99 | 5.01 | 1.84 | 4.46 | 2.12 | 4.67 | 1.98 |
| **Reproductive information**  e.g. fertility information, miscarriage, abortion | 4.42 | 2.01 | 4.93 | 1.76 | 4.20 | 2.14 | 4.93 | 1.91 | 4.40 | 2.07 | 4.62 | 1.96 |
| **Genetic information**  e.g. genetic test information, paternity test | 4.41 | 2.09 | 4.94 | 1.92 | 4.25 | 2.09 | 4.78 | 1.96 | 4.41 | 2.12 | 4.46 | 2.08 |
| **Social activity & media usage**  e.g. frequency social contacts, amount of media usage | 4.57 | 1.88 | 4.70 | 1.90 | 4.26 | 2.08 | 4.71 | 1.99 | 4.24 | 2.15 | 4.28 | 2.06 |

**Appendix A (cont.). Variables for Management of Personal Information (Studies 1-3)**

| **Items** | **Study 1** | | | | **Study 2** | | | | **Study 3** | | | |
| --- | --- | --- | --- | --- | --- | --- | --- | --- | --- | --- | --- | --- |
|  | **PC** | | **UH** | | **PC** | | **UH** | | **PC** | | **UH** | |
| **Self-disclosure intentions.** Please specify the extent to which you would be willing to reveal the following personal information**:** | **M** | **SD** | **M** | **SD** | **M** | **SD** | **M** | **SD** | **M** | **SD** | **M** | **SD** |

| **Contact information**  e.g. address, phone number, ZIP code | 4.29 | 1.96 | 4.94 | 1.80 | 4.06 | 2.22 | 4.81 | 1.90 | 4.35 | 2.03 | 4.52 | 1.96 |
| --- | --- | --- | --- | --- | --- | --- | --- | --- | --- | --- | --- | --- |
| **Shopping habits**  e.g. frequency, location, time | 4.43 | 2.00 | 4.68 | 1.78 | 4.37 | 2.08 | 4.72 | 2.01 | 4.37 | 2.06 | 4.30 | 1.96 |
| **Sensory data**  e.g. mobile device, wearables | 4.19 | 1.92 | 4.65 | 1.86 | 4.05 | 2.13 | 4.67 | 2.00 | 3.98 | 2.07 | 4.25 | 2.11 |
| **Location data**  e.g. GPS, Bluetooth | 3.99 | 2.08 | 4.14 | 2.12 | 3.85 | 2.13 | 4.17 | 2.06 | 3.74 | 2.12 | 3.71 | 2.11 |
| **Falsification intentions.**^1^ Please specify the extent to which you would want to manage your personal information in the following way: | **M** | **SD** | **M** | **SD** | **M** | **SD** | **M** | **SD** | **M** | **SD** | **M** | **SD** |
| I am likely to give false information | 3.16 | 2.04 | 2.95 | 2.15 | 2.97 | 1.95 | 3.03 | 2.09 | 3.04 | 2.03 | 3.06 | 2.06 |
| I purposely try to trick when providing my personal data | 3.07 | 1.98 | 3.03 | 1.99 | 2.97 | 1.93 | 3.07 | 2.01 | 3.05 | 2.00 | 3.18 | 2.13 |
| I think it is fine to give misleading answers on personal questions | 3.08 | 2.02 | 3.12 | 2.15 | 2.98 | 1.88 | 3.25 | 2.18 | 3.12 | 2.05 | 3.20 | 2.09 |

Notes. The items referred to either the MWC General Hospital or MWC Pharma depending on the experimental condition; M = Mean; SD = Standard deviation; unstandardized values are shown; PC = Pharmaceutical company; UH = University hospital. For self-disclosure intentions items, individuals indicated their willingness to reveal personal information of the different categories on a seven-point rating scale, with response options ranging from 1 = very unlikely to 7 = very likely. For falsification intentions items, individuals indicated their willingness to falsify personal information on a seven-point rating scale, with response options ranging from 1 = very unlikely to 7 = very likely. ^1^ We eliminated one item to improve the validity and reliability of the variable (i.e., I would only fill up data partially [34]).

**Appendix B. Variables for Motive Perception (Studies 1-3)**

| **Items** When you think about potential reasons why XX is collecting your personal data to set up this database, how much do you agree with the following explanations? XX is collecting my personal data to set up a database because they … | **Study 1** | | | | **Study 2** | | | | **Study 3** | | | |
| --- | --- | --- | --- | --- | --- | --- | --- | --- | --- | --- | --- | --- |
|  | **PC** | | **UH** | | **PC** | | **UH** | | **PC** | | **UH** | |
| **Altruistic motives** | **M** | **SD** | **M** | **SD** | **M** | **SD** | **M** | **SD** | **M** | **SD** | **M** | **SD** |
| Ultimately care about people | 4.90 | 1.57 | 5.32 | 1.28 | 4.54 | 1.53 | 5.39 | 1.12 | 4.66 | 1.59 | 5.04 | 1.49 |
| Have a genuine concern for the welfare of people | 4.93 | 1.50 | 5.43 | 1.19 | 4.74 | 1.53 | 5.45 | 1.09 | 4.74 | 1.68 | 5.21 | 1.38 |
| Really care about getting health information to people | 4.99 | 1.42 | 5.38 | 1.31 | 4.84 | 1.58 | 5.34 | 1.10 | 4.82 | 1.66 | 5.21 | 1.36 |
| Want to help people to help | 5.06 | 1.39 | 5.36 | 1.31 | 4.86 | 1.47 | 5.38 | 1.14 | 4.82 | 1.56 | 5.24 | 1.31 |
| Believe it is morally the “right” thing to do | 4.86 | 1.46 | 5.05 | 1.44 | 4.64 | 1.57 | 5.32 | 1.30 | 4.65 | 1.66 | 4.91 | 1.52 |
| Have a long-terms interest in the community | 4.98 | 1.44 | 5.30 | 1.30 | 4.86 | 1.53 | 5.35 | 1.17 | 4.71 | 1.61 | 5.10 | 1.45 |
| Are trying to give back something to the community | 4.82 | 1.63 | 5.16 | 1.30 | 4.59 | 1.62 | 5.14 | 1.27 | 4.66 | 1.66 | 4.95 | 1.57 |
| Want to make it easier for people who care about the cause to support it | 5.18 | 1.30 | 5.24 | 1.19 | 4.82 | 1.44 | 5.31 | 1.23 | 4.87 | 1.56 | 4.89 | 1.54 |
| **Egoistic motives**^1^ | **M** | **SD** | **M** | **SD** | **M** | **SD** | **M** | **SD** | **M** | **SD** | **M** | **SD** |
| Want to get publicity | 4.73 | 1.59 | 4.41 | 1.56 | 4.83 | 1.49 | 4.52 | 1.71 | 4.96 | 1.45 | 4.35 | 1.74 |
| Are taking advantage of the cause to help their own business | 4.74 | 1.68 | 4.34 | 1.71 | 4.84 | 1.59 | 4.33 | 1.80 | 4.82 | 1.58 | 4.44 | 1.77 |
| Want to help themselves | 5.16 | 1.33 | 4.84 | 1.44 | 5.22 | 1.38 | 4.61 | 1.60 | 5.12 | 1.48 | 4.68 | 1.64 |
| Will keep more customers by making this offer | 4.81 | 1.49 | 4.41 | 1.54 | 4.78 | 1.47 | 4.71 | 1.58 | 4.72 | 1.37 | 4.66 | 1.74 |
| Will get more customers by making this offer | 4.82 | 1.55 | 4.43 | 1.56 | 4.99 | 1.42 | 4.56 | 1.65 | 4.84 | 1.45 | 4.49 | 1.71 |
| Hope to increase profits by making this offer | 5.02 | 1.50 | 4.54 | 1.64 | 5.11 | 1.47 | 4.74 | 1.69 | 5.24 | 1.43 | 4.56 | 1.75 |

Notes. XX in the item was substituted with either the MWC General Hospital or MWC Pharma depending on the experimental condition; M = Mean; SD = Standard deviation; unstandardized values are shown; PC = Pharmaceutical company; UH = University hospital. Participants indicated their level of agreement to the statements on a seven-point rating scale from 1 = I strongly disagree to 7 = I strongly agree. ^1^ We eliminated two items to improve the validity and reliability of the variable (i.e., Want to affect what people think about them [69]; Believe it creates a positive corporate image [58]).

**Appendix C. Summary Statistics Data (Study 1): Means, Standard Deviations, and Correlations (Square Root of the Average Variance Extracted)**

| **Variable** | **M** | **SD** | **1** | **2** | **3** | **4** | **5** |
| --- | --- | --- | --- | --- | --- | --- | --- |
| 1 Information requester | 0.50 | 0.50 | – |  |  |  |  |
| 2 Altruistic motives (parallel mediator 1) | 5.11 | 1.17 | -.14 | .73 |  |  |  |
| 3 Egoistic motives (parallel mediator 2) | 4.69 | 1.21 | .16 | -.07 | .78 |  |  |
| 4 Self-disclosure intentions (outcome 1) | 5.03 | 1.15 | -.18 | .64 | -.00 | .66 |  |
| 5 Falsification intentions (outcome 2) | 3.30 | 1.80 | .02 | .07 | .46 | .07 | .90 |

Notes. n = 204. Information requester was coded 0 for the university hospital and 1 for the pharmaceutical company. Cell sizes: n = 103 for the university hospital, n = 101 for the pharmaceutical company. Seven-point rating scales from (lowest value) to 7 (highest value) were used. Prior to the confirmatory factor and path analysis, items were standardized. The altruistic motives variable is the average of eight standardized items, the egoistic motives variable is the average of six standardized items, the self-disclosure intentions variable is the average of 20 standardized items, and the falsification intentions variable is the average of three standardized items. The square root of the average variance extracted is shown in the diagonal and correlations are shown below the diagonal.

**Appendix D. Summary Statistics Data (Study 2): Means, Standard Deviations, and Correlations (Square Root of the Average Variance Extracted)**

| **Variable** | **M** | **SD** | **1** | **2** | **3** | **4** | **5** | **6** |
| --- | --- | --- | --- | --- | --- | --- | --- | --- |
| 1 Information requester | 0.49 | 0.50 | – |  |  |  |  |  |
| 2 Message appeal | 0.50 | 0.50 | .00 | – |  |  |  |  |
| 3 Altruistic motives (parallel mediator 1) | 5.04 | 1.16 | -.26 | .07 | .80 |  |  |  |
| 4 Egoistic motives (parallel mediator 2) | 4.80 | 1.17 | .16 | -.01 | -.03 | .71 |  |  |
| 5 Self-disclosure intentions (outcome 1) | 4.80 | 1.45 | -.21 | -.04 | .70 | .03 | .75 |  |
| 6 Falsification intentions (outcome 2) | 3.05 | 1.89 | -.04 | -.00 | -.04 | .34 | .03 | .91 |

Notes. n = 330. Information requester was coded 0 for the university hospital and 1 for the pharmaceutical company; message appeal was coded 0 for others-benefit and 1 for self-benefit. Cell sizes: n = 83 for the university hospital and others-benefit condition, n = 84 for the university hospital and self-benefit condition, n = 81 for the pharmaceutical company and others-benefit condition, and n = 82 for the pharmaceutical company and self-benefit condition. Prior to the confirmatory factor and path analysis, items were standardized. The altruistic motives variable is the average of eight standardized items, the egoistic motives variable is the average of six standardized items, the self-disclosure intentions variable is the average of 20 standardized items, and the falsification intentions variable is the average of three standardized items. The square root of the average variance extracted is shown in the diagonal and correlations are shown below the diagonal.

**Appendix E. Summary Statistics Data (Study 3): Means, Standard Deviations, and Correlations (Square Root of the Average Variance Extracted)**

| **Variable** | **M** | **SD** | **1** | **2** | **3** | **4** | **5** | **6** |
| --- | --- | --- | --- | --- | --- | --- | --- | --- |
| 1 Information requester | 0.50 | 0.50 | – |  |  |  |  |  |
| 2 Message endorser | 0.50 | 0.50 | -.04 | – |  |  |  |  |
| 3 Altruistic motives (parallel mediator 1) | 4.91 | 1.28 | -.13 | .02 | .80 |  |  |  |
| 4 Egoistic motives (parallel mediator 2) | 4.75 | 1.17 | .17 | -.02 | -.01 | .71 |  |  |
| 5 Self-disclosure intentions (outcome 1) | 4.68 | 1.48 | -.07 | -.03 | .70 | .00 | .75 |  |
| 6 Falsification intentions (outcome 2) | 3.11 | 1.94 | -.02 | .07 | .11 | .43 | .13 | .92 |

Notes. n = 330. Information requester was coded 0 for the university hospital and 1 for the pharmaceutical company; credibility of the message endorser was coded 0 for high (Birx) and 1 for low (Trump). Cell sizes: n = 78 for the university hospital and high credibility condition, n = 86 for the university hospital and low credibility condition, n = 85 for the pharmaceutical company and high credibility condition, and n = 79 for the pharmaceutical company and low credibility condition. Prior to the confirmatory factor and path analysis, items were standardized. The altruistic motives variable is the average of eight standardized items, the egoistic motives variable is the average of six standardized items, the self-disclosure intentions variable is the average of 20 standardized items, and the falsification intentions variable is the average of three standardized items. The square root of the average variance extracted is shown in the diagonal and correlations are shown below the diagonal.

**Appendix F. Hypothetical Scenario Provided to the Participants (Studies 1-3)**

First, please read the hypothetical scenario carefully and picture yourself in the specified circumstances.

Given the current situation with the COVID-19 virus the university hospital MWC General Hospital [the pharmaceutical company MWC Pharma] announced that they are setting up a comprehensive database of people’s health data to make better predictions about the virus, to improve current and future management of the virus, and to support people with recommendations on how to cope with their daily health challenges.

The MWC General Hospital [MWC Pharma] is known to be committed to leading the revolution in healthcare through cutting edge technology. It aims to expand medical knowledge and advance health and well-being.

To address the evolving needs of patients, they make use of their superior competences in artificial intelligence across various areas. To meet their goals and to ensure that the outcomes are as precise as possible, the MWC General Hospital [MWC Pharma] requires a large amount of health data. On this basis they call upon the general population to contribute to this database

To do that, you access the secure website of the MWC General Hospital [MWC Pharma]. Here, you will be asked about which kind of personal data you would be willing to provide. The data does not only refer to virus-related health patterns, but focuses on predictors of peoples’ likelihood to be infected, to recover, and to be treated accordingly.

Notes. The items referred to either the MWC General Hospital or MWC Pharma depending on the experimental condition.

**Appendix G. Manipulation Text for the Two Message Appeal Conditions (Study 2)**

**Others-benefit**

Empower PEOPLE’S health, share data! Data sharing can transform healthcare and provides benefits to all people. With the help of data sharing, you will directly enhance people’s lives. Everyone will make better informed decisions. Everyone will get a better treatment. This will increase well-being of all people.

**Self-benefit**

Empower YOUR health, share data! Data sharing can transform healthcare and provides benefits to you personally. With the help of data sharing, you will directly enhance your quality of life. You will make better informed decisions. You will get a better treatment. This will increase your well-being.

**Appendix H. Manipulation Text for the Two Message Endorser Credibility Conditions (Study 3)**

Recently, U.S. President Donald Trump [U.S. Representative for Global Health Diplomacy Deborah Birx, M.D.,] has encouraged people to provide personal health data to support organizations like the the MWC General Hospital [MWC Pharma] in their efforts. In a recent press conference, President Trump [M.D. Birx] highlighted the importance of up-to-date and real health data as supporting information for experts in health and medicine.

**Appendix I. Variables for Credibility and Domain-specific Expertise (Study 3)**

| **Items** | **DB** | | | | **DT** | | | | | |
| --- | --- | --- | --- | --- | --- | --- | --- | --- | --- | --- |
|  | **PC** | | **UH** | | **PC** | | **UH** | | |  |
| **Credibility.** How much do you agree with the following statements. XX is .... | **M** | **SD** | **M** | **SD** | **M** | **SD** | **M** | **SD** |  |  |
| Dependable | 5.01 | 1.34 | 4.82 | 1.54 | 3.76 | 2.23 | 4.11 | 2.21 |  |  |
| Honest | 4.97 | 1.57 | 4.81 | 1.54 | 3.70 | 2.21 | 4.18 | 2.29 |  |  |
| Reliable | 5.14 | 1.37 | 4.89 | 1.59 | 3.74 | 2.32 | 4.16 | 2.20 |  |  |
| Sincere | 5.03 | 1.41 | 4.92 | 1.56 | 3.99 | 2.22 | 4.08 | 2.18 |  |  |
| Trustworthy | 4.05 | 1.47 | 4.93 | 1.60 | 3.69 | 2.26 | 3.97 | 2.16 |  |  |
| Expert | 2.18 | 1.13 | 5.28 | 1.59 | 3.69 | 2.25 | 3.87 | 2.19 |  |  |
| Experienced | 5.54 | 1.15 | 5.49 | 1.40 | 4.02 | 2.18 | 4.30 | 2.20 |  |  |
| Knowledgeable | 5.63 | 1.11 | 5.35 | 1.37 | 3.91 | 2.28 | 4.38 | 2.13 |  |  |
| Qualified | 5.71 | 1.24 | 5.34 | 1.34 | 3.88 | 2.21 | 4.27 | 2.21 |  |  |
| Skilled | 5.40 | 1.39 | 5.29 | 1.43 | 3.93 | 2.18 | 4.23 | 2.06 |  |  |
| Friendly | 5.04 | 1.46 | 4.89 | 1.46 | 3.74 | 2.03 | 4.18 | 2.01 |  |  |
| Likeable | 4.94 | 1.38 | 4.96 | 1.46 | 3.77 | 2.09 | 4.11 | 2.21 |  |  |
| Warm | 4.94 | 1.38 | 4.87 | 1.49 | 3.74 | 2.26 | 3.99 | 2.12 |  |  |
| Approachable | 5.12 | 1.34 | 4.95 | 1.53 | 3.94 | 2.21 | 4.05 | 2.18 |  |  |
| **Domain Expertise.** With regards to Covid-19, I would consider XX to be … | **M** | **SD** | **M** | **SD** | **M** | **SD** | **M** | **SD** |  |  |
| An expert | 5.60 | 1.23 | 5.16 | 1.51 | 3.67 | 2.34 | 3.84 | 2.29 |  |  |
| Experienced | 5.60 | 1.13 | 5.42 | 1.55 | 3.58 | 2.22 | 4.00 | 2.27 |  |  |
| Knowledgeable | 5.47 | 1.17 | 5.44 | 1.34 | 3.67 | 2.22 | 3.95 | 2.17 |  |  |
| Qualified | 5.55 | 1.16 | 5.47 | 1.32 | 3.59 | 2.21 | 3.91 | 2.21 |  |  |
| Skilled | 5.62 | 1.17 | 5.44 | 1.33 | 3.58 | 2.16 | 3.96 | 2.22 |  |  |

Notes. XX in the item was substituted with either President Donald Trump or M.D. Deborah Birx depending on the experimental condition; M = Mean; SD = Standard Deviation; unstandardized values are shown; DB = Deborah Birx; DT = Donald Trump; PC = Pharmaceutical company; UH = University hospital.
