# Supplementary material for: Motive perception pathways to the release of personal information to healthcare organizations
Source: BMC Med Inform Decis Mak. 2022 Sep 13;22:240. doi: 10.1186/s12911-022-01986-4 (PMC9468521; doi:10.1186/s12911-022-01986-4)
Supplement: Supplementary file 2 — Additional file 2. Questionnaire for Study 1. [file 12911_2022_1986_MOESM2_ESM.docx]

Questionnaire for Study 1

Intro

Thank you very much for participating in this survey conducted by [University].

On the following pages we will ask you about your attitudes towards the provision of personal information and health data to health care providers.

There is no right or wrong answer, so please give us your best indication of how you would decide in the given situation.

The survey will take approximately 8-10 minutes to complete.

Your data and results will be treated and evaluated confidentially. All data will be anonymized and only used for scientific purposes.

Participation in this survey is voluntary and you can choose to not participate or exit the survey at any times

Thank you very much for your participation!

Consent

By selecting "I consent" you state that you have read the foregoing information and consent to voluntarily participate in this research.

- I consent (1)
- I do not consent (2)

captcha

Please check the box below to start the survey.

hospital

First, please read the hypothetical scenario on the next page carefully and picture yourself in the specified circumstances.

Given the current situation with the COVID-19 virus the university hospital “MWC General Hospital” announced that they are setting up a comprehensive database of people’s health data to make better predictions about the virus, to improve current and future management of the virus, and to support people with recommendations on how to cope with their daily health challenges

MWC General Hospital” is known to be committed to leading the revolution in healthcare through cutting edge technology. It aims to expand medical knowledge and advance health and well-being

To address the evolving needs of patients they make use of their superior competences in artificial intelligence across various areas.

To meet their goals and to ensure that the outcomes are as precise as possible, “MWC General Hospital” requires a large amount of health data. On this basis they call upon the general population to contribute to this database

Timing

To do that, you access the secure website of “MWC General Hospital”. Here, you will be asked about which kind of personal data you would be willing to provide.

The data does not only refer to virus-related health patterns, but focuses on predictors of peoples’ likelihood to be infected, to recover and to be treated accordingly.

pharma

First, please read the hypothetical scenario on the next page carefully and picture yourself in the specified circumstances.

Given the current situation with the COVID-19 virus the pharmaceutical company “MWC Pharma” announced that they are setting up a comprehensive database of people’s health data to make better predictions about the virus, to improve current and future management of the virus, and to support people with recommendations on how to cope with their daily health challenges.

MWC Pharma” is known to be committed to leading the revolution in healthcare through cutting edge technology. It aims to expand medical knowledge and advance health and well-being.

To address the evolving needs of patients they make use of their superior competences in artificial intelligence across various areas.

To meet their goals and to ensure that the outcomes are as precise as possible, “MWC Pharma” requires a large amount of health data. On this basis they call upon the general population to contribute to this database.

Timing

To do that, you access the secure website of “MWC Pharma”. Here, you will be asked about which kind of personal data you would be willing to provide.

The data does not only refer to virus-related health patterns, but focuses on predictors of peoples’ likelihood to be infected, to recover and to be treated accordingly

Disclosure

To set up the database, "MWC General Hospital" is asking for some of your personal data.

Please specify the extent to which you would be willing to reveal personal information of the following categories:

|  | Very unlikely (1) | Moderately unlikely (2) | Slightly unlikely (3) | Neither likely nor unlikely (4) | Slightly likely (5) | Moderately likely (6) | Very likely (7) |
| --- | --- | --- | --- | --- | --- | --- | --- |
| Sexual health information (e.g. sexual transmitted diseases, HIV) (1) |  |  |  |  |  |  |  |
| Mental health information (e.g. psychological therapy or counseling, depression, anxiety disorder, suicide attempts, eating disorder) (2) |  |  |  |  |  |  |  |
| Substance abuse (e.g. drug, medication or alcohol abuse) (3) |  |  |  |  |  |  |  |
| Emotional information (e.g. happiness, sadness, fear) (4) |  |  |  |  |  |  |  |
| Contact information (e.g. address, phone number, ZIP code) (5) |  |  |  |  |  |  |  |
| Reproductive information (e.g. fertility information, miscarriage, abortion) (6) |  |  |  |  |  |  |  |
| Social activity & media usage (e.g. frequency social contacts, amount of media usage) (7) |  |  |  |  |  |  |  |
| Genetic information (e.g. genetic test information, paternity test (8) |  |  |  |  |  |  |  |
| Sensory data (e.g. mobile device, wearables) (9) |  |  |  |  |  |  |  |
| Location data (e.g. GPS, Bluetooth) (10) |  |  |  |  |  |  |  |
| Medical history (e.g. previous illnesses, injuries, surgeries) (11) |  |  |  |  |  |  |  |
| Current health condition (e.g. symptoms, illnesses, chronic disease, hospitalization) (12) |  |  |  |  |  |  |  |
| Lifestyle (e.g. amount of leisure time, working hours, mobility/transportation behavior) (13) |  |  |  |  |  |  |  |
| Test results (e.g. blood pressure, cholesterol level, screening results, mammogram results) (14) |  |  |  |  |  |  |  |
| General health (e.g. smoking habits, BMI, physical activity, diet, weight, sleeping habits, alcohol consumption) (15) |  |  |  |  |  |  |  |
| Medication (e.g. prescribed mediation, over the counter medication, medication adherence) (16) |  |  |  |  |  |  |  |
| Surgery (e.g. transplants, plastic surgery, hip replacement) (17) |  |  |  |  |  |  |  |
| Demographics (e.g. age, gender) (18) |  |  |  |  |  |  |  |
| Occupation (19) |  |  |  |  |  |  |  |
| Shopping habits (e.g. frequency, location, time) (20) |  |  |  |  |  |  |  |

falsify

|  |  |
| --- | --- |

Think about you provide your personal information in this given situation.

Please indicate in how far you agree with the following statements:

|  | Strongly disagree (1) | Disagree (2) | Somewhat disagree (3) | Neither agree nor disagree (4) | Somewhat agree (5) | Agree (6) | Strongly agree (7) |
| --- | --- | --- | --- | --- | --- | --- | --- |
| I am likely to give false information (1) |  |  |  |  |  |  |  |
| I purposely try to trick when providing my personal data (2) |  |  |  |  |  |  |  |
| I think it is fine to give misleading answers on personal questions (3) |  |  |  |  |  |  |  |
| I would only fill up data partially (5) |  |  |  |  |  |  |  |

|  |  |
| --- | --- |

**attributed motives**

When you think about potential reasons why “MWC General Hospital” is collecting your personal data to set up this database, how much do you agree with the following explanations? The university hospital is collecting my personal data to set up a database, because they...

|  | Strongly disagree (1) | Disagree (2) | Somewhat disagree (3) | Neither agree nor disagree (4) | Somewhat agree (5) | Agree (6) | Strongly agree (7) |
| --- | --- | --- | --- | --- | --- | --- | --- |
| ...ultimately care about people (97) |  |  |  |  |  |  |  |
| ...have a genuine concern for the welfare of people (98) |  |  |  |  |  |  |  |
| ...really care about getting health information to people (99) |  |  |  |  |  |  |  |
| ...want to help people to help (100) |  |  |  |  |  |  |  |
| ...believe it is morally the “right” thing to do (101) |  |  |  |  |  |  |  |
| ...have a long-terms interest in the community (102) |  |  |  |  |  |  |  |
| ...are trying to give back something to the community (103) |  |  |  |  |  |  |  |
| ...want to make it easier for people who care about the cause to support it (104) |  |  |  |  |  |  |  |
| ...want to get publicity (105) |  |  |  |  |  |  |  |
| ...are taking advantage of the cause to help their own business (106) |  |  |  |  |  |  |  |
| ...want to affect what people think about them (107) |  |  |  |  |  |  |  |
| ...want to help themselves (108) |  |  |  |  |  |  |  |
| ...will keep more customers by making this offer (109) |  |  |  |  |  |  |  |
| ...will get more customers by making this offer (110) |  |  |  |  |  |  |  |
| ...hope to increase profits by making this offer (111) |  |  |  |  |  |  |  |
| ...believe it creates a positive corporate image (112) |  |  |  |  |  |  |  |

sensitivity

|  |
| --- |

Please indicate how sensitive the personal information in each of the following categories is to you on a level of: "1 = not sensitive at all" to "7 = very sensitive":

|  | Not sensitive at all (1) | (2) | (3) | Neither nor (4) | (5) | (6) | Very sensitive (7) |
| --- | --- | --- | --- | --- | --- | --- | --- |
| Sexual health information (e.g. sexual transmitted diseases, HIV) (1) |  |  |  |  |  |  |  |
| Mental health information (e.g. psychological therapy or counseling, depression, anxiety disorder, suicide attempts, eating disorder) (2) |  |  |  |  |  |  |  |
| Substance abuse (e.g. drug, medication or alcohol abuse) (3) |  |  |  |  |  |  |  |
| Emotional information (e.g. happiness, sadness, fear) (4) |  |  |  |  |  |  |  |
| Contact information (e.g. address, phone number, ZIP code) (5) |  |  |  |  |  |  |  |
| Reproductive information (e.g. fertility information, miscarriage, abortion) (6) |  |  |  |  |  |  |  |
| Social activity & media usage (e.g. frequency social contacts, amount of media usage) (7) |  |  |  |  |  |  |  |
| Genetic information (e.g. genetic test information, paternity test (8) |  |  |  |  |  |  |  |
| Sensory data (e.g. mobile device, wearables) (9) |  |  |  |  |  |  |  |
| Location data (e.g. GPS, Bluetooth) (10) |  |  |  |  |  |  |  |
| Medical history (e.g. previous illnesses, injuries, surgeries) (11) |  |  |  |  |  |  |  |
| Current health condition (e.g. symptoms, illnesses, chronic disease, hospitalization) (12) |  |  |  |  |  |  |  |
| Lifestyle (e.g. amount of leisure time, working hours, mobility/transportation behavior) (13) |  |  |  |  |  |  |  |
| Test results (e.g. blood pressure, cholesterol level, screening results, mammogram results) (14) |  |  |  |  |  |  |  |
| General health (e.g. smoking habits, BMI, physical activity, diet, weight, sleeping habits, alcohol consumption) (15) |  |  |  |  |  |  |  |
| Medication (e.g. prescribed mediation, over the counter medication, medication adherence) (16) |  |  |  |  |  |  |  |
| Surgery (e.g. transplants, plastic surgery, hip replacement) (17) |  |  |  |  |  |  |  |
| Demographics (e.g. age, gender) (18) |  |  |  |  |  |  |  |
| Occupation (19) |  |  |  |  |  |  |  |
| Shopping habits (e.g. frequency, location, time) (20) |  |  |  |  |  |  |  |

benefit individual

|  |  |
| --- | --- |

Please rate the extent to which you feel that you would personally benefit from providing your personal information to “MWC General Hospital” for their database:

|  | (1) | (2) | (3) | (4) | (5) | (6) | (7) |  |
| --- | --- | --- | --- | --- | --- | --- | --- | --- |
| No benefit |  |  |  |  |  |  |  | Great benefit |

|  |  |
| --- | --- |

How much do you agree with the following statements?

|  | Strongly disagree (1) | Disagree (2) | Somewhat disagree (3) | Neither agree nor disagree (4) | Somewhat agree (5) | Agree (6) | Strongly agree (7) |
| --- | --- | --- | --- | --- | --- | --- | --- |
| I will receive value from the ways the hospital uses my personal data (1) |  |  |  |  |  |  |  |
| I value how my personal information is used to customize my experience (2) |  |  |  |  |  |  |  |
| I need to provide my personal information so I can get exactly what I want from the hospital's database (3) |  |  |  |  |  |  |  |
| I believe that as a result of my personal information disclosure, I will benefit from a better, customized service and/or better information and products (4) |  |  |  |  |  |  |  |
| Providing my personal information to the hospital entails benefits to me (5) |  |  |  |  |  |  |  |

benefit society

|  |  |
| --- | --- |

Please rate the extent to which you feel that the society benefits from providing your personal information to “MWC General Hospital” for their database:

|  | (1) | (2) | (3) | (4) | (5) | (6) | (7) |  |
| --- | --- | --- | --- | --- | --- | --- | --- | --- |
| No benefit |  |  |  |  |  |  |  | Great benefit |

How much do you agree with the following statements?

|  | Strongly disagree (1) | Disagree (2) | Somewhat disagree (3) | Neither agree nor disagree (4) | Somewhat agree (5) | Agree (6) | Strongly agree (7) |
| --- | --- | --- | --- | --- | --- | --- | --- |
| I believe that others/ society receive value from the way the hospital uses my personal data (1) |  |  |  |  |  |  |  |
| I need to provide my personal information so that also others/ society can benefit from the hospital's database (2) |  |  |  |  |  |  |  |
| By collecting my personal details, the hospital improves the well-being of others/society (3) |  |  |  |  |  |  |  |
| I believe that as a result of my personal information disclosure, others/society will benefit from a better, customized service and/or better information and products (4) |  |  |  |  |  |  |  |
| Providing my personal information to the hospital entails benefits to others/society (5) |  |  |  |  |  |  |  |

control

|  |  |
| --- | --- |

Please indicate how much you agree to the following statements that describe your expected control over your personal data.

I expect to have...

|  | Strongly disagree (1) | Disagree (2) | Somewhat disagree (3) | Neither agree nor disagree (4) | Somewhat agree (5) | Agree (6) | Strongly agree (7) |
| --- | --- | --- | --- | --- | --- | --- | --- |
| ...control over how the hospital uses my personal information (1) |  |  |  |  |  |  |  |
| ...control over whether my personal information is shared with others (2) |  |  |  |  |  |  |  |
| ...access to the personal information collected about me by the hospital (3) |  |  |  |  |  |  |  |
| ...the ability to edit the personal information collected about me by the hospital (4) |  |  |  |  |  |  |  |

involvement

When you think about the COVID-19 virus, how important is this topic to you?

The cause ....

|  | (1) | (2) | (3) | (4) | (5) | (6) | (7) |  |
| --- | --- | --- | --- | --- | --- | --- | --- | --- |
| Is unimportant to me |  |  |  |  |  |  |  | Is important to me |
| Means nothing to me |  |  |  |  |  |  |  | Means a lot to me |
| Is personally irrelevant to me |  |  |  |  |  |  |  | Is personally relevant to me |
| Doesn't matter a great deal to me |  |  |  |  |  |  |  | Matters a great deal to me |

Fairness

How fair would you consider the exchange between you and “MWC General Hospital”?

Please specify your level of agreement with the following statements:

|  | Strongly disagree (1) | Disagree (2) | Somewhat disagree (3) | Neither agree nor disagree (4) | Somewhat agree (5) | Agree (6) | Strongly agree (7) |
| --- | --- | --- | --- | --- | --- | --- | --- |
| What I give up in terms of releasing my personal information to the hospital is commensurate with what I will receive in return (1) |  |  |  |  |  |  |  |
| Given the potential problems of releasing my personal information to the hospital, the benefits I will receive from the hospital are fair (2) |  |  |  |  |  |  |  |
| I will be fairly rewarded for providing personal information to the hospital (3) |  |  |  |  |  |  |  |
| I feel that the outcome I will receive for providing personal information to the hospital is fair (4) |  |  |  |  |  |  |  |

How fair do you consider the process of the information exchange with “MWC General Hospital”?

Please specify your level of agreement with the following statements:

|  | Strongly disagree (1) | Disagree (2) | Somewhat disagree (3) | Neither agree nor disagree (4) | Somewhat agree (5) | Agree (6) | Strongly agree (7) |
| --- | --- | --- | --- | --- | --- | --- | --- |
| I believe their use of my personal information is fair (1) |  |  |  |  |  |  |  |
| I believe the hospital accesses my information in a fair way (2) |  |  |  |  |  |  |  |
| I believe the hospital’s use of my information is ethical (3) |  |  |  |  |  |  |  |
| I believe the hospital manages my information in an equitable way (4) |  |  |  |  |  |  |  |
| I believe the hospital has fair policies and practices to handle problems (5) |  |  |  |  |  |  |  |

privacy concern

Now think about your personal information that you should provide to the database of "MWC General Hospital". Please specify your level of agreement with the following statements:

|  | Strongly disagree (1) | Disagree (2) | Somewhat disagree (3) | Neither agree nor disagree (4) | Somewhat agree (5) | Agree (6) | Strongly agree (7) |
| --- | --- | --- | --- | --- | --- | --- | --- |
| I am concerned that the information I submit to the hospital could be misused (1) |  |  |  |  |  |  |  |
| I am concerned that others can find private information about me from the hospital (2) |  |  |  |  |  |  |  |
| I am concerned about providing personal information to the hospital, because it could be used in a way I did not foresee (3) |  |  |  |  |  |  |  |
| It bothers me when the hospital asks me for this much personal information (4) |  |  |  |  |  |  |  |
| I am concerned that the hospital is collecting too much personal information about me (5) |  |  |  |  |  |  |  |
| I believe it is important that I am aware of and have knowledgeable about how the hospital would use personal information that I had disclosed (6) |  |  |  |  |  |  |  |
| I am concerned that unauthorized people may access my personal information (7) |  |  |  |  |  |  |  |
| I am concerned that the hospital may keep my personal information in a non-accurate manner (8) |  |  |  |  |  |  |  |
| I believe the hospital should disclose reasons for wanting my personal information (9) |  |  |  |  |  |  |  |

relevance

Would you consider the information asked by “MWC General Hospital” as relevant for this cause?

|  | 1 (1) | 2 (2) | 3 (3) | 4 (4) | 5 (5) | 6 (6) | 7 (7) |  |
| --- | --- | --- | --- | --- | --- | --- | --- | --- |
| Not relevant at all |  |  |  |  |  |  |  | Extremely relevant |

Did you expect “MWC General Hospital” to ask you for this set of information for this cause?

|  | 1 (1) | 2 (2) | 3 (3) | 4 (4) | 5 (5) | 6 (6) | 7 (7) |  |
| --- | --- | --- | --- | --- | --- | --- | --- | --- |
| Not expected at all |  |  |  |  |  |  |  | Very much expected |

**Covid-19 sensitivity**

|  |
| --- |

Below, you find a list of factors that are important in the context of COVID-19.

Finally, please specify how sensitive the following personal data are to you.

|  | Not sensitive at all (1) | (2) | (3) | Neither nor (4) | (5) | (6) | Very sensitive (7) |
| --- | --- | --- | --- | --- | --- | --- | --- |
| Data tracing your general movements (1) |  |  |  |  |  |  |  |
| Data tracing your social interactions (2) |  |  |  |  |  |  |  |
| Data tracing your shopping habits (3) |  |  |  |  |  |  |  |
| Gender information (4) |  |  |  |  |  |  |  |
| Travel history (5) |  |  |  |  |  |  |  |
| Age (6) |  |  |  |  |  |  |  |
| ZIP code (7) |  |  |  |  |  |  |  |
| Smoking (8) |  |  |  |  |  |  |  |
| History of your respiratory diseases (9) |  |  |  |  |  |  |  |
| History of your cardiac diseases (10) |  |  |  |  |  |  |  |
| Body mass index (based on weight and height) (11) |  |  |  |  |  |  |  |

demographics

|  |
| --- |

age

How old are you?

________________________________________________________________

Please indicate your gender:

- Female (1)
- Male (2)
- Other (3)

Please indicate the size of the household you are currently living in:

- 1 person (1)
- 2 persons (2)
- 3 persons (3)
- 4 persons (4)
- more than 4 persons (5)

Please indicate your highest degree of education:

- Less than high school (11)
- High school graduate (12)
- Some college (13)
- Bachelor's degree (14)
- Master's degree (15)
- Professional degree (16)
- Doctorate (17)

Please select the U.S. state in which you are currently living in.

- Alabama (64)
- Alaska (65)
- Arizona (66)
- Arkansas (67)
- California (68)
- Colorado (69)
- Connecticut (70)
- Delaware (71)
- Florida (72)
- Georgia (73)
- Hawaii (74)
- Idaho (75)
- Illinois (76)
- Indiana (77)
- Iowa (78)
- Kansas (79)
- Kentucky (80)
- Louisiana (81)
- Maine (82)
- Maryland (83)
- Massachusetts (84)
- Michigan (85)
- Minnesota (86)
- Mississippi (87)
- Missouri (88)
- Montana (89)
- Nebraska (90)
- Nevada (91)
- New Hampshire (92)
- New Jersey (93)
- New Mexico (94)
- New York (95)
- North Carolina (96)
- North Dakota (97)
- Ohio (98)
- Oklahoma (99)
- Oregon (100)
- Pennsylvania (101)
- Rhode Island (102)
- South Carolina (103)
- South Dakota (104)
- Tennessee (105)
- Texas (106)
- Utah (107)
- Vermont (108)
- Virginia (109)
- Washington (110)
- West Virginia (111)
- Wisconsin (112)
- Wyoming (113)

Do you own a smartphone

- Yes (1)
- No (2)

In general, would you say your health is?

|  | Poor (1) | Fair (2) | Good (3) | Very good (4) | Excellent (5) |
| --- | --- | --- | --- | --- | --- |
| In general I would say my health is.... (1) |  |  |  |  |  |

How high do you perceive the risk of being infected by COVID-19 yourself?

|  | 1 (1) | 2 (2) | 3 (3) | 4 (4) | 5 (5) | 6 (6) | 7 (7) |  |
| --- | --- | --- | --- | --- | --- | --- | --- | --- |
| Very low risk |  |  |  |  |  |  |  | Very high risk |

How high do you perceive the risk that COVID-19 negatively affects your health?

|  | 1 (1) | 2 (2) | 3 (3) | 4 (4) | 5 (5) | 6 (6) | 7 (7) |  |
| --- | --- | --- | --- | --- | --- | --- | --- | --- |
| Very low risk |  |  |  |  |  |  |  | Very high risk |

Would you like to leave us a comment?

________________________________________________________________

End
