# Supplementary material for: Motive perception pathways to the release of personal information to healthcare organizations
Source: BMC Med Inform Decis Mak. 2022 Sep 13;22:240. doi: 10.1186/s12911-022-01986-4 (PMC9468521; doi:10.1186/s12911-022-01986-4)
Supplement: Supplementary file 4 — Additional file 4. Questionnaire for Study 3. [file 12911_2022_1986_MOESM4_ESM.docx]

Questionnaire for Study 3

Start of Block: Intro

Thank you very much for participating in this survey conducted by [University]. The purpose of this study is to understand your attitudes towards the provision of personal information and health data to health care providers. You will read about a hypothetical scenario in the health context, and then be asked about your opinion on this topic. There is no right or wrong answer, so please give us your best indication of how you would respond in the given situation. The survey will take approximately 12-14 minutes to complete, and you will be compensated with $ 1 for your participation. You will receive payment to your account via the MTurk validation code we will provide you at the end of the survey. You must enter this validation code to receive payment. Your data and results will be treated and evaluated confidentially. All data will be anonymized and only used for scientific purposes. Participation in this survey is completely voluntary and you may choose to not participate or exit the survey at any times. Thank you very much for your participation!

End of Block: Intro

Start of Block: Consent

I agree to participate in this research. I have been fully informed about the nature and purpose of this study, and I fully understand my involvement as a participant. I understand that any data I provide will be used for no other purpose than the study, and details I provide will be kept confidential. I understand I have the right to withdraw from the study at any time before completing the end of the questionnaire. By selecting "I consent" you state that you have read the foregoing information and consent to voluntarily participate in this research.

- I consent
- I do not consent

End of Block: Consent

Start of Block: captcha

Please check the box below to start the survey.

End of Block: captcha

Start of Block: hospital_Trump

First, please read the hypothetical scenario on the next page carefully and picture yourself in the specified circumstances.

| Page Break |  |
| --- | --- |

Given the current situation with the COVID-19 virus the university hospital “MWC General Hospital” announced that they are setting up a comprehensive database of people’s health data to make better predictions about the virus, to expedite the search for a cure, and to support people with recommendations on how to cope with their daily health challenges. “MWC General Hospital” is known to be committed to leading the revolution in healthcare through cutting edge technology. It aims to expand medical knowledge and advance health and well-being. To address the evolving needs of patients they make use of their superior competences in artificial intelligence across various areas.

| Page Break |  |
| --- | --- |

To meet their goals and to ensure that the outcomes are as precise as possible, “MWC General Hospital” requires a large amount of health data. On this basis they call upon the general population to contribute to this database. Recently, U.S. President Donald Trump, has encouraged people to provide personal health data to support organizations like the “MWC General Hospital” in their efforts. In a recent press conference, President Trump highlighted the importance of up-to-date and real health data as supporting information for experts in health and medicine.

Timing

First Click

Last Click

Page Submit

Click Count

| Page Break |  |
| --- | --- |

To do that, you access the secure website of “MWC General Hospital”. Here, you will be asked about which kind of personal data you would be willing to provide. The data does not only refer to virus-related health patterns, but focuses on predictors of peoples’ likelihood to be infected, to recover and to be treated accordingly.

| Page Break |  |
| --- | --- |

End of Block: hospital_Trump

Start of Block: disclosure

| 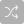 | 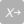 |
| --- | --- |

To set up the database, "MWC General Hospital" is asking for some of your personal data. Please specify the extent to which you would be willing to reveal personal information of the following categories:

|  | Very unlikely | Moderately unlikely | Slightly unlikely | Neither likely nor unlikely | Slightly likely | Moderately likely | Very likely |
| --- | --- | --- | --- | --- | --- | --- | --- |
| Sexual health information (e.g. sexual transmitted diseases, HIV) |  |  |  |  |  |  |  |
| Mental health information (e.g. psychological therapy or counseling, depression, anxiety disorder, suicide attempts, eating disorder) |  |  |  |  |  |  |  |
| Substance abuse (e.g. drug, medication or alcohol abuse) |  |  |  |  |  |  |  |
| Emotional information (e.g. happiness, sadness, fear) |  |  |  |  |  |  |  |
| Contact information (e.g. address, phone number, ZIP code) |  |  |  |  |  |  |  |
| Reproductive information (e.g. fertility information, miscarriage, abortion) |  |  |  |  |  |  |  |
| Social activity & media usage (e.g. frequency social contacts, amount of media usage) |  |  |  |  |  |  |  |
| Genetic information (e.g. genetic test information, paternity test |  |  |  |  |  |  |  |
| Sensory data (e.g. mobile device, wearables) |  |  |  |  |  |  |  |
| Location data (e.g. GPS, Bluetooth) |  |  |  |  |  |  |  |
| Medical history (e.g. previous illnesses, injuries, surgeries) |  |  |  |  |  |  |  |
| Current health condition (e.g. symptoms, illnesses, chronic disease, hospitalization) |  |  |  |  |  |  |  |
| Lifestyle (e.g. amount of leisure time, working hours, mobility/transportation behavior) |  |  |  |  |  |  |  |
| Test results (e.g. blood pressure, cholesterol level, screening results, mammogram results) |  |  |  |  |  |  |  |
| General health (e.g. smoking habits, BMI, physical activity, diet, weight, sleeping habits, alcohol consumption) |  |  |  |  |  |  |  |
| Medication (e.g. prescribed mediation, over the counter medication, medication adherence) |  |  |  |  |  |  |  |
| Surgery (e.g. transplants, plastic surgery, hip replacement) |  |  |  |  |  |  |  |
| Demographics (e.g. age, gender) |  |  |  |  |  |  |  |
| Occupation |  |  |  |  |  |  |  |
| Shopping habits (e.g. frequency, location, time) |  |  |  |  |  |  |  |

End of Block: disclosure

Start of Block: falsify

| 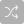 | 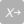 |
| --- | --- |

Think about how you provide your personal information to "MWC General Hospital". Please indicate in how far you agree with the following statements:

|  | Strongly disagree | Disagree | Somewhat disagree | Neither agree nor disagree | Somewhat agree | Agree | Strongly agree |
| --- | --- | --- | --- | --- | --- | --- | --- |
| I am likely to give false information |  |  |  |  |  |  |  |
| I purposely try to trick when providing my personal data |  |  |  |  |  |  |  |
| I think it is fine to give misleading answers on personal questions |  |  |  |  |  |  |  |
| I would only fill up data partially |  |  |  |  |  |  |  |

End of Block: falsify

Start of Block: attributed motives

| 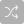 | 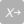 |
| --- | --- |

When you think about potential reasons why “MWC General Hospital” is collecting your personal data to set up this database, how much do you agree with the following explanations? The university hospital is collecting my personal data to set up a database, because they...

|  | Strongly disagree | Disagree | Somewhat disagree | Neither agree nor disagree | Somewhat agree | Agree | Strongly agree |
| --- | --- | --- | --- | --- | --- | --- | --- |
| ...ultimately care about people |  |  |  |  |  |  |  |
| ...have a genuine concern for the welfare of people |  |  |  |  |  |  |  |
| ...really care about getting health information to people |  |  |  |  |  |  |  |
| ...want to help people to help others |  |  |  |  |  |  |  |
| ...believe it is morally the “right” thing to do |  |  |  |  |  |  |  |
| ...have a long-terms interest in the community |  |  |  |  |  |  |  |
| ...are trying to give back something to the community |  |  |  |  |  |  |  |
| ...want to make it easier for people who care about the cause to support it |  |  |  |  |  |  |  |
| ...want to get publicity |  |  |  |  |  |  |  |
| ...are taking advantage of the cause to help their own business |  |  |  |  |  |  |  |
| ...want to affect what people think about them |  |  |  |  |  |  |  |
| ...want to help themselves |  |  |  |  |  |  |  |
| ...will keep more customers by making this offer |  |  |  |  |  |  |  |
| ...will get more customers by making this offer |  |  |  |  |  |  |  |
| ...hope to increase profits by making this offer |  |  |  |  |  |  |  |
| ...believe it creates a positive corporate image |  |  |  |  |  |  |  |

End of Block: attributed motives

Start of Block: sensitivity

| 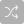 |
| --- |

Please indicate how sensitive the personal information in each of the following categories is to you on a level of: " 1 = not sensitive at all" to " 7 = very sensitive" :

|  | Not sensitive at all |  |  | Neither nor |  |  | Very sensitive |
| --- | --- | --- | --- | --- | --- | --- | --- |
| Sexual health information (e.g. sexual transmitted diseases, HIV) |  |  |  |  |  |  |  |
| Mental health information (e.g. psychological therapy or counseling, depression, anxiety disorder, suicide attempts, eating disorder) |  |  |  |  |  |  |  |
| Substance abuse (e.g. drug, medication or alcohol abuse) |  |  |  |  |  |  |  |
| Emotional information (e.g. happiness, sadness, fear) |  |  |  |  |  |  |  |
| Contact information (e.g. address, phone number, ZIP code) |  |  |  |  |  |  |  |
| Reproductive information (e.g. fertility information, miscarriage, abortion) |  |  |  |  |  |  |  |
| Social activity & media usage (e.g. frequency social contacts, amount of media usage) |  |  |  |  |  |  |  |
| Genetic information (e.g. genetic test information, paternity test |  |  |  |  |  |  |  |
| Sensory data (e.g. mobile device, wearables) |  |  |  |  |  |  |  |
| Location data (e.g. GPS, Bluetooth) |  |  |  |  |  |  |  |
| Medical history (e.g. previous illnesses, injuries, surgeries) |  |  |  |  |  |  |  |
| Current health condition (e.g. symptoms, illnesses, chronic disease, hospitalization) |  |  |  |  |  |  |  |
| Lifestyle (e.g. amount of leisure time, working hours, mobility/transportation behavior) |  |  |  |  |  |  |  |
| Test results (e.g. blood pressure, cholesterol level, screening results, mammogram results) |  |  |  |  |  |  |  |
| General health (e.g. smoking habits, BMI, physical activity, diet, weight, sleeping habits, alcohol consumption) |  |  |  |  |  |  |  |
| Medication (e.g. prescribed mediation, over the counter medication, medication adherence) |  |  |  |  |  |  |  |
| Surgery (e.g. transplants, plastic surgery, hip replacement) |  |  |  |  |  |  |  |
| Demographics (e.g. age, gender) |  |  |  |  |  |  |  |
| Occupation |  |  |  |  |  |  |  |
| Shopping habits (e.g. frequency, location, time) |  |  |  |  |  |  |  |

End of Block: sensitivity

Start of Block: benefit individual

| 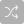 | 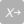 |
| --- | --- |

Please rate the extent to which you feel that you would personally benefit from providing your personal information to “MWC General Hospital” for their database:

|  |  |  |  |  |  |  |  |  |
| --- | --- | --- | --- | --- | --- | --- | --- | --- |
| No benefit |  |  |  |  |  |  |  | Great benefit |

| 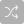 | 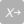 |
| --- | --- |

How much do you agree with the following statements?

|  | Strongly disagree | Disagree | Somewhat disagree | Neither agree nor disagree | Somewhat agree | Agree | Strongly agree |
| --- | --- | --- | --- | --- | --- | --- | --- |
| I will receive value from the ways the hospital uses my personal data |  |  |  |  |  |  |  |
| I value how my personal information is used to customize my experience |  |  |  |  |  |  |  |
| I need to provide my personal information so I can get exactly what I want from the hospital's database |  |  |  |  |  |  |  |
| I believe that as a result of my personal information disclosure, I will benefit from a better, customized service and/or better information and products |  |  |  |  |  |  |  |
| Providing my personal information to the hospital entails benefits to me |  |  |  |  |  |  |  |

End of Block: benefit individual

Start of Block: benefit society

| 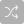 | 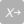 |
| --- | --- |

Please rate the extent to which you feel that the society benefits from providing your personal information to “MWC General Hospital” for their database:

|  |  |  |  |  |  |  |  |  |
| --- | --- | --- | --- | --- | --- | --- | --- | --- |
| No benefit |  |  |  |  |  |  |  | Great benefit |

| 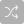 | 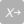 |
| --- | --- |

How much do you agree with the following statements?

|  | Strongly disagree | Disagree | Somewhat disagree | Neither agree nor disagree | Somewhat agree | Agree | Strongly agree |
| --- | --- | --- | --- | --- | --- | --- | --- |
| I believe that others/ society receive value from the way the hospital uses my personal data |  |  |  |  |  |  |  |
| I need to provide my personal information so that also others/ society can benefit from the hospital's database |  |  |  |  |  |  |  |
| By collecting my personal details, the hospital improves the well-being of others/society |  |  |  |  |  |  |  |
| I believe that as a result of my personal information disclosure, others/society will benefit from a better, customized service and/or better information and products |  |  |  |  |  |  |  |
| Providing my personal information to the hospital entails benefits to others/society |  |  |  |  |  |  |  |

End of Block: benefit society

Start of Block: control

| 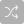 | 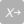 |
| --- | --- |

Please indicate how much you agree to the following statements that describe the level of control you wish to have over your personal data. I expect/wish to have...

|  | Strongly disagree | Disagree | Somewhat disagree | Neither agree nor disagree | Somewhat agree | Agree | Strongly agree |
| --- | --- | --- | --- | --- | --- | --- | --- |
| ...control over how the hospital uses my personal information |  |  |  |  |  |  |  |
| ...control over whether my personal information is shared with others |  |  |  |  |  |  |  |
| ...access to the personal information collected about me by the hospital |  |  |  |  |  |  |  |
| ...the ability to edit the personal information collected about me by the hospital |  |  |  |  |  |  |  |

End of Block: control

Start of Block: involvement

| 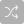 | 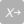 |
| --- | --- |

When you think about the COVID-19 virus, how important is this topic to you? The cause ....

|  |  |  |  |  |  |  |  |  |
| --- | --- | --- | --- | --- | --- | --- | --- | --- |
| Is unimportant to me |  |  |  |  |  |  |  | Is important to me |
| Means nothing to me |  |  |  |  |  |  |  | Means a lot to me |
| Is personally irrelevant to me |  |  |  |  |  |  |  | Is personally relevant to me |
| Doesn't matter a great deal to me |  |  |  |  |  |  |  | Matters a great deal to me |

End of Block: involvement

Start of Block: fairness

| 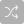 | 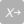 |
| --- | --- |

How fair would you consider the exchange between you and “MWC General Hospital”? Please specify your level of agreement with the following statements:

|  | Strongly disagree | Disagree | Somewhat disagree | Neither agree nor disagree | Somewhat agree | Agree | Strongly agree |
| --- | --- | --- | --- | --- | --- | --- | --- |
| What I give up in terms of releasing my personal information to the hospital is commensurate with what I will receive in return |  |  |  |  |  |  |  |
| Given the potential problems of releasing my personal information to the hospital, the benefits I will receive from the hospital are fair |  |  |  |  |  |  |  |
| I will be fairly rewarded for providing personal information to the hospital |  |  |  |  |  |  |  |
| I feel that the outcome I will receive for providing personal information to the hospital is fair |  |  |  |  |  |  |  |

| Page Break |  |
| --- | --- |

| 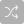 | 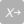 |
| --- | --- |

How fair do you consider the process of the information exchange with “MWC General Hospital”? Please specify your level of agreement with the following statements:

|  | Strongly disagree | Disagree | Somewhat disagree | Neither agree nor disagree | Somewhat agree | Agree | Strongly agree |
| --- | --- | --- | --- | --- | --- | --- | --- |
| I believe their use of my personal information is fair |  |  |  |  |  |  |  |
| I believe the hospital accesses my information in a fair way |  |  |  |  |  |  |  |
| I believe the hospital’s use of my information is ethical |  |  |  |  |  |  |  |
| I believe the hospital manages my information in an equitable way |  |  |  |  |  |  |  |
| I believe the hospital has fair policies and practices to handle problems |  |  |  |  |  |  |  |

| Page Break |  |
| --- | --- |

End of Block: fairness

Start of Block: privacy concern

| 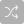 | 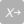 |
| --- | --- |

Now think about the information that you should provide to the database of "MWC General Hospital". Please specify your level of agreement with the following statements regarding your concern towards your personal information: I am concerned...

|  | Strongly disagree | Disagree | Somewhat disagree | Neither agree nor disagree | Somewhat agree | Agree | Strongly agree |
| --- | --- | --- | --- | --- | --- | --- | --- |
| ...that the information I submit to the hospital could be misused |  |  |  |  |  |  |  |
| ...that others can find private information about me from the hospital |  |  |  |  |  |  |  |
| ...about providing personal information to the hospital, because it could be used in a way I did not foresee |  |  |  |  |  |  |  |
| ...that the hospital is collecting too much personal information about me |  |  |  |  |  |  |  |
| ...about providing personal to the hospital, because of what others might do with it |  |  |  |  |  |  |  |
| ...that unauthorized people may access my personal information |  |  |  |  |  |  |  |
| ...that the hospital may keep my personal information in a non-accurate manner |  |  |  |  |  |  |  |

End of Block: privacy concern

Start of Block: source credibility_ Trump

| 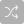 |
| --- |

How much do you agree with the following statements. President Donald Trump is...

|  | Strongly disagree | Somewhat disagree | Disagree | Neither agree nor disagree | Somewhat agree | Agree | Strongly agree |
| --- | --- | --- | --- | --- | --- | --- | --- |
| Dependable |  |  |  |  |  |  |  |
| Honest |  |  |  |  |  |  |  |
| Reliable |  |  |  |  |  |  |  |
| Sincere |  |  |  |  |  |  |  |
| Trustworthy |  |  |  |  |  |  |  |
| Expert |  |  |  |  |  |  |  |
| Experienced |  |  |  |  |  |  |  |
| Knowledgeable |  |  |  |  |  |  |  |
| Qualified |  |  |  |  |  |  |  |
| Skilled |  |  |  |  |  |  |  |
| Friendly |  |  |  |  |  |  |  |
| Likeable |  |  |  |  |  |  |  |
| Warm |  |  |  |  |  |  |  |
| Approachable |  |  |  |  |  |  |  |

End of Block: source credibility_ Trump

Start of Block: domain expertise_Trump

| 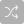 |
| --- |

In your opinion, how much do the below statements apply to President Donald Trump with regards to the COVID-19 virus? Please specify your level of agreement. With regards to COVID-19, I would consider President Donald Trump to be…

|  | Strongly Disagree | Disagree | Somewhat disagree | Neither agree nor disagree | Somewhat agree | Agree | Strongly agree |
| --- | --- | --- | --- | --- | --- | --- | --- |
| Expert |  |  |  |  |  |  |  |
| Experienced |  |  |  |  |  |  |  |
| Knowledgeable |  |  |  |  |  |  |  |
| Qualified |  |  |  |  |  |  |  |
| Skilled |  |  |  |  |  |  |  |

End of Block: domain expertise_Trump

Start of Block: orientation_Trump

| 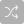 |
| --- |

Please indicate below, how much you agree with the following statements. President Donald Trump is genuinely interested in...

|  | Strongly Disagree | Disagree | Somewhat disagree | Neither agree nor disagree | Somewhat agree | Agree | Strongly agree |
| --- | --- | --- | --- | --- | --- | --- | --- |
| Promoting the U.S. economy |  |  |  |  |  |  |  |
| Boosting the U.S. job market |  |  |  |  |  |  |  |
| Stimulating the U.S. stock exchange |  |  |  |  |  |  |  |
| Promoting public health in the U.S. |  |  |  |  |  |  |  |
| Fighting inequalities in access to health in the U.S. |  |  |  |  |  |  |  |
| Preventing disease to spread out in the U. S. |  |  |  |  |  |  |  |

End of Block: orientation_Trump

Start of Block: hospital_Birx

First, please read the hypothetical scenario on the next page carefully and picture yourself in the specified circumstances.

| Page Break |  |
| --- | --- |

Given the current situation with the COVID-19 virus the university hospital “MWC General Hospital” announced that they are setting up a comprehensive database of people’s health data to make better predictions about the virus, to expedite the search for a cure, and to support people with recommendations on how to cope with their daily health challenges. “MWC General Hospital” is known to be committed to leading the revolution in healthcare through cutting edge technology. It aims to expand medical knowledge and advance health and well-being. To address the evolving needs of patients they make use of their superior competences in artificial intelligence across various areas.

| Page Break |  |
| --- | --- |

To meet their goals and to ensure that the outcomes are as precise as possible, “MWC General Hospital” requires a large amount of health data. On this basis they call upon the general population to contribute to this database. Recently, U.S. Representative for Global Health Diplomacy Deborah Birx, M.D., has encouraged people to provide personal health data to support organizations like the “MWC General Hospital” in their efforts. In a recent press conference, M.D. Birx highlighted the importance of up-to-date and real health data as supporting information for experts in health and medicine.

Timing

First Click

Last Click

Page Submit

Click Count

| Page Break |  |
| --- | --- |

To do that, you access the secure website of “MWC General Hospital”. Here, you will be asked about which kind of personal data you would be willing to provide. The data does not only refer to virus-related health patterns, but focuses on predictors of peoples’ likelihood to be infected, to recover and to be treated accordingly.

| Page Break |  |
| --- | --- |

End of Block: hospital_Birx

Start of Block: source credibility_Birx

| 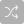 |
| --- |

How much do you agree with the following statements. M.D. Deborah Birx is...

|  | Strongly disagree | Somewhat disagree | Disagree | Neither agree nor disagree | Somewhat agree | Agree | Strongly agree |
| --- | --- | --- | --- | --- | --- | --- | --- |
| Dependable |  |  |  |  |  |  |  |
| Honest |  |  |  |  |  |  |  |
| Reliable |  |  |  |  |  |  |  |
| Sincere |  |  |  |  |  |  |  |
| Trustworthy |  |  |  |  |  |  |  |
| Expert |  |  |  |  |  |  |  |
| Experienced |  |  |  |  |  |  |  |
| Knowledgeable |  |  |  |  |  |  |  |
| Qualified |  |  |  |  |  |  |  |
| Skilled |  |  |  |  |  |  |  |
| Friendly |  |  |  |  |  |  |  |
| Likeable |  |  |  |  |  |  |  |
| Warm |  |  |  |  |  |  |  |
| Approachable |  |  |  |  |  |  |  |

End of Block: source credibility_Birx

Start of Block: domain expertise_Birx

| 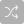 |
| --- |

In your opinion, how much do the below statements apply to M.D. Deborah Birx with regards to the COVID-19 virus ? Please specify your level of agreement. With regards to COVID-19, I would consider M.D. Deborah Birx to be…

|  | Strongly Disagree | Disagree | Somewhat disagree | Neither agree nor disagree | Somewhat agree | Agree | Strongly agree |
| --- | --- | --- | --- | --- | --- | --- | --- |
| Expert |  |  |  |  |  |  |  |
| Experienced |  |  |  |  |  |  |  |
| Knowledgeable |  |  |  |  |  |  |  |
| Qualified |  |  |  |  |  |  |  |
| Skilled |  |  |  |  |  |  |  |

End of Block: domain expertise_Birx

Start of Block: orientation_Birx

| 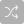 |
| --- |

Please indicate below, how much you agree with the following statements. M.D. Deborah Birx is genuinely interested in ...

|  | Strongly Disagree | Disagree | Somewhat disagree | Neither agree nor disagree | Somewhat agree | Agree | Strongly agree |
| --- | --- | --- | --- | --- | --- | --- | --- |
| Promoting the U.S. economy |  |  |  |  |  |  |  |
| Boosting the U.S. job market |  |  |  |  |  |  |  |
| Stimulating the U.S. stock exchange |  |  |  |  |  |  |  |
| Promoting public health in the U.S. |  |  |  |  |  |  |  |
| Fighting inequalities in access to health in the U.S. |  |  |  |  |  |  |  |
| Preventing disease to spread out in the U. S. |  |  |  |  |  |  |  |

End of Block: orientation_Birx

Start of Block: pharma_Trump

First, please read the hypothetical scenario on the next page carefully and picture yourself in the specified circumstances.

| Page Break |  |
| --- | --- |

Given the current situation with the COVID-19 virus the pharmaceutical company “MWC Pharma” announced that they are setting up a comprehensive database of people’s health data to make better predictions about the virus, to improve current and future management of the virus, and to support people with recommendations on how to cope with their daily health challenges. “MWC Pharma” is known to be committed to leading the revolution in healthcare through cutting edge technology. It aims to expand medical knowledge and advance health and well-being. To address the evolving needs of patients they make use of their superior competences in artificial intelligence across various areas.

| Page Break |  |
| --- | --- |

To meet their goals and to ensure that the outcomes are as precise as possible, “MWC Pharma” requires a large amount of health data. On this basis they call upon the general population to contribute to this database. Recently, U.S. President Donald Trump, has encouraged people to provide personal health data to support organizations like “MWC Pharma” in their efforts. In a recent press conference, President Trump highlighted the importance of up-to-date and real health data as supporting information for experts in health and medicine.

Timing

First Click

Last Click

Page Submit

Click Count

| Page Break |  |
| --- | --- |

To do that, you access the secure website of “MWC Pharma”. Here, you will be asked about which kind of personal data you would be willing to provide. The data does not only refer to virus-related health patterns, but focuses on predictors of peoples’ likelihood to be infected, to recover and to be treated accordingly.

| Page Break |  |
| --- | --- |

End of Block: pharma_Trump

Start of Block: disclosure

| 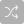 | 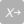 |
| --- | --- |

To set up the database, “MWC Pharma” is asking for some of your personal data. Please specify the extent to which you would be willing to reveal personal information of the following categories:

|  | Very unlikely | Moderately unlikely | Slightly unlikely | Neither likely nor unlikely | Slightly likely | Moderately likely | Very likely |
| --- | --- | --- | --- | --- | --- | --- | --- |
| Sexual health information (e.g. sexual transmitted diseases, HIV) |  |  |  |  |  |  |  |
| Mental health information (e.g. psychological therapy or counseling, depression, anxiety disorder, suicide attempts, eating disorder) |  |  |  |  |  |  |  |
| Substance abuse (e.g. drug, medication or alcohol abuse) |  |  |  |  |  |  |  |
| Emotional information (e.g. happiness, sadness, fear) |  |  |  |  |  |  |  |
| Contact information (e.g. address, phone number, ZIP code) |  |  |  |  |  |  |  |
| Reproductive information (e.g. fertility information, miscarriage, abortion) |  |  |  |  |  |  |  |
| Social activity & media usage (e.g. frequency social contacts, amount of media usage) |  |  |  |  |  |  |  |
| Genetic information (e.g. genetic test information, paternity test |  |  |  |  |  |  |  |
| Sensory data (e.g. mobile device, wearables) |  |  |  |  |  |  |  |
| Location data (e.g. GPS, Bluetooth) |  |  |  |  |  |  |  |
| Medical history (e.g. previous illnesses, injuries, surgeries) |  |  |  |  |  |  |  |
| Current health condition (e.g. symptoms, illnesses, chronic disease, hospitalization) |  |  |  |  |  |  |  |
| Lifestyle (e.g. amount of leisure time, working hours, mobility/transportation behavior) |  |  |  |  |  |  |  |
| Test results (e.g. blood pressure, cholesterol level, screening results, mammogram results) |  |  |  |  |  |  |  |
| General health (e.g. smoking habits, BMI, physical activity, diet, weight, sleeping habits, alcohol consumption) |  |  |  |  |  |  |  |
| Medication (e.g. prescribed mediation, over the counter medication, medication adherence) |  |  |  |  |  |  |  |
| Surgery (e.g. transplants, plastic surgery, hip replacement) |  |  |  |  |  |  |  |
| Demographics (e.g. age, gender) |  |  |  |  |  |  |  |
| Occupation |  |  |  |  |  |  |  |
| Shopping habits (e.g. frequency, location, time) |  |  |  |  |  |  |  |

End of Block: disclosure

Start of Block: falsify pharma

| 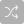 | 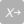 |
| --- | --- |

Think about how you provide your personal information to "MWC Pharma". Please indicate in how far you agree with the following statements:

|  | Strongly disagree | Disagree | Somewhat disagree | Neither agree nor disagree | Somewhat agree | Agree | Strongly agree |
| --- | --- | --- | --- | --- | --- | --- | --- |
| I am likely to give false information |  |  |  |  |  |  |  |
| I purposely try to trick when providing my personal data |  |  |  |  |  |  |  |
| I think it is fine to give misleading answers on personal questions |  |  |  |  |  |  |  |
| I would only fill up data partially |  |  |  |  |  |  |  |

End of Block: falsify pharma

Start of Block: attributed motives

| 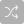 | 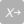 |
| --- | --- |

When you think about potential reasons why “MWC Pharma” is collecting your personal data to set up this database, how much do you agree with the following explanations? The pharmaceutical company is collecting my personal data to set up a database, because they...

|  | Strongly disagree | Disagree | Somewhat disagree | Neither agree nor disagree | Somewhat agree | Agree | Strongly agree |
| --- | --- | --- | --- | --- | --- | --- | --- |
| ...ultimately care about people |  |  |  |  |  |  |  |
| ...have a genuine concern for the welfare of people |  |  |  |  |  |  |  |
| ...really care about getting health information to people |  |  |  |  |  |  |  |
| ...want to help people to help others |  |  |  |  |  |  |  |
| ...believe it is morally the “right” thing to do |  |  |  |  |  |  |  |
| ...have a long-terms interest in the community |  |  |  |  |  |  |  |
| ...are trying to give back something to the community |  |  |  |  |  |  |  |
| ...want to make it easier for people who care about the cause to support it |  |  |  |  |  |  |  |
| ...want to get publicity |  |  |  |  |  |  |  |
| ...are taking advantage of the cause to help their own business |  |  |  |  |  |  |  |
| ...want to affect what people think about them |  |  |  |  |  |  |  |
| ...want to help themselves |  |  |  |  |  |  |  |
| ...will keep more customers by making this offer |  |  |  |  |  |  |  |
| ...will get more customers by making this offer |  |  |  |  |  |  |  |
| ...hope to increase profits by making this offer |  |  |  |  |  |  |  |
| ...believe it creates a positive corporate image |  |  |  |  |  |  |  |

End of Block: attributed motives

Start of Block: benefit individual

| 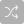 | 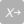 |
| --- | --- |

Please rate the extent to which you feel that you would personally benefit from providing your personal information to “MWC Pharma” for their database:

|  |  |  |  |  |  |  |  |  |
| --- | --- | --- | --- | --- | --- | --- | --- | --- |
| No benefit |  |  |  |  |  |  |  | Great benefit |

| 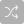 | 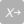 |
| --- | --- |

How much do you agree with the following statements?

|  | Strongly disagree | Disagree | Somewhat disagree | Neither agree nor disagree | Somewhat agree | Agree | Strongly agree |
| --- | --- | --- | --- | --- | --- | --- | --- |
| I will receive value from the ways the pharmaceutical company uses my personal data |  |  |  |  |  |  |  |
| I value how my personal information is used to customize my experience |  |  |  |  |  |  |  |
| I need to provide my personal information so I can get exactly what I want from the pharmaceutical company's database |  |  |  |  |  |  |  |
| I believe that as a result of my personal information disclosure, I will benefit from a better, customized service and/or better information and products |  |  |  |  |  |  |  |
| Providing my personal information to the pharmaceutical company entails benefits to me |  |  |  |  |  |  |  |

End of Block: benefit individual

Start of Block: benefit society

| 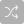 | 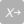 |
| --- | --- |

Please rate the extent to which you feel that the society benefits from providing your personal information to “MWC Pharma” for their database:

|  |  |  |  |  |  |  |  |  |
| --- | --- | --- | --- | --- | --- | --- | --- | --- |
| No benefit |  |  |  |  |  |  |  | Great benefit |

| 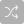 | 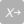 |
| --- | --- |

How much do you agree with the following statements?

|  | Strongly disagree | Disagree | Somewhat disagree | Neither agree nor disagree | Somewhat agree | Agree | Strongly agree |
| --- | --- | --- | --- | --- | --- | --- | --- |
| I believe that others/ society receive value from the way the pharmaceutical company uses my personal data |  |  |  |  |  |  |  |
| I need to provide my personal information so that also others/ society can benefit from the pharmaceutical company's database |  |  |  |  |  |  |  |
| By collecting my personal details, the pharmaceutical company improves the well-being of others/society |  |  |  |  |  |  |  |
| I believe that as a result of my personal information disclosure, others/society will benefit from a better, customized service and/or better information and products |  |  |  |  |  |  |  |
| Providing my personal information to the pharmaceutical company entails benefits to others/society |  |  |  |  |  |  |  |

End of Block: benefit society

Start of Block: control

| 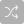 | 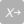 |
| --- | --- |

Please indicate how much you agree to the following statements that describe the level of control you wish to have over your personal data. I expect/wish to have...

|  | Strongly disagree | Disagree | Somewhat disagree | Neither agree nor disagree | Somewhat agree | Agree | Strongly agree |
| --- | --- | --- | --- | --- | --- | --- | --- |
| ...control over how the pharmaceutical company uses my personal information |  |  |  |  |  |  |  |
| ...control over whether my personal information is shared with others |  |  |  |  |  |  |  |
| ...access to the personal information collected about me by the pharmaceutical company |  |  |  |  |  |  |  |
| ...the ability to edit the personal information collected about me by the pharmaceutical company |  |  |  |  |  |  |  |

End of Block: control

Start of Block: fairness

| 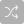 | 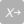 |
| --- | --- |

How fair would you consider the exchange between you and “MWC Pharma”? Please specify your level of agreement with the following statements:

|  | Strongly disagree | Disagree | Somewhat disagree | Neither agree nor disagree | Somewhat agree | Agree | Strongly agree |
| --- | --- | --- | --- | --- | --- | --- | --- |
| What I give up in terms of releasing my personal information to the pharmaceutical company is commensurate with what I will receive in return |  |  |  |  |  |  |  |
| Given the potential problems of releasing my personal information to the pharmaceutical company, the benefits I will receive from the hospital are fair |  |  |  |  |  |  |  |
| I will be fairly rewarded for providing personal information to the pharmaceutical company |  |  |  |  |  |  |  |
| I feel that the outcome I will receive for providing personal information to the pharmaceutical company is fair |  |  |  |  |  |  |  |

| Page Break |  |
| --- | --- |

| 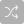 | 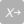 |
| --- | --- |

How fair do you consider the process of the information exchange with “MWC Pharma”? Please specify your level of agreement with the following statements:

|  | Strongly disagree | Disagree | Somewhat disagree | Neither agree nor disagree | Somewhat agree | Agree | Strongly agree |
| --- | --- | --- | --- | --- | --- | --- | --- |
| I believe their use of my personal information is fair |  |  |  |  |  |  |  |
| I believe the pharmaceutical company accesses my information in a fair way |  |  |  |  |  |  |  |
| I believe the pharmaceutical company’s use of my information is ethical |  |  |  |  |  |  |  |
| I believe the pharmaceutical company manages my information in an equitable way |  |  |  |  |  |  |  |
| I believe the pharmaceutical company has fair policies and practices to handle problems |  |  |  |  |  |  |  |

| Page Break |  |
| --- | --- |

End of Block: fairness

Start of Block: privacy concern

| 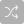 | 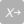 |
| --- | --- |

Now think about the information that you should provide to the database of "MWC Pharma". Please specify your level of agreement with the following statements regarding your concern towards your personal information: I am concerned...

|  | Strongly disagree | Disagree | Somewhat disagree | Neither agree nor disagree | Somewhat agree | Agree | Strongly agree |
| --- | --- | --- | --- | --- | --- | --- | --- |
| ...that the information I submit to the pharmaceutical company could be misused |  |  |  |  |  |  |  |
| ...that others can find private information about me from pharmaceutical company |  |  |  |  |  |  |  |
| ...about providing personal information to the pharmaceutical company, because it could be used in a way I did not foresee |  |  |  |  |  |  |  |
| ...that the pharmaceutical company is collecting too much personal information about me |  |  |  |  |  |  |  |
| ...that unauthorized people may access my personal information |  |  |  |  |  |  |  |
| ...that the pharmaceutical company may keep my personal information in a non-accurate manner |  |  |  |  |  |  |  |
| ...about providing personal to the pharmaceutical company, because of what others might do with it |  |  |  |  |  |  |  |

End of Block: privacy concern

Start of Block: pharma_Birx

First, please read the hypothetical scenario on the next page carefully and picture yourself in the specified circumstances.

| Page Break |  |
| --- | --- |

Given the current situation with the COVID-19 virus the pharmaceutical company “MWC Pharma” announced that they are setting up a comprehensive database of people’s health data to make better predictions about the virus, to improve current and future management of the virus, and to support people with recommendations on how to cope with their daily health challenges. “MWC Pharma” is known to be committed to leading the revolution in healthcare through cutting edge technology. It aims to expand medical knowledge and advance health and well-being. To address the evolving needs of patients they make use of their superior competences in artificial intelligence across various areas.

| Page Break |  |
| --- | --- |

To meet their goals and to ensure that the outcomes are as precise as possible, “MWC Pharma” requires a large amount of health data. On this basis they call upon the general population to contribute to this database. Recently, U.S. Representative for Global Health Diplomacy Deborah Birx, M.D., has encouraged people to provide personal health data to support organizations like “MWC Pharma” in their efforts. In a recent press conference, M.D. Birx highlighted the importance of up-to-date and real health data as supporting information for experts in health and medicine.

Timing

First Click

Last Click

Page Submit

Click Count

| Page Break |  |
| --- | --- |

To do that, you access the secure website of “MWC Pharma”. Here, you will be asked about which kind of personal data you would be willing to provide. The data does not only refer to virus-related health patterns, but focuses on predictors of peoples’ likelihood to be infected, to recover and to be treated accordingly.

| Page Break |  |
| --- | --- |

End of Block: pharma_Birx

Start of Block: Covid-19 sensitivity

| 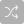 |
| --- |

Below, you find a list of factors that are important in the context of COVID-19. Finally, please specify how sensitive the following personal data are to you.

|  | Not sensitive at all |  |  | Neither nor |  |  | Very sensitive |
| --- | --- | --- | --- | --- | --- | --- | --- |
| Data tracing your general movements |  |  |  |  |  |  |  |
| Data tracing your social interactions |  |  |  |  |  |  |  |
| Data tracing your shopping habits |  |  |  |  |  |  |  |
| Gender information |  |  |  |  |  |  |  |
| Travel history |  |  |  |  |  |  |  |
| Age |  |  |  |  |  |  |  |
| ZIP code |  |  |  |  |  |  |  |
| Smoking |  |  |  |  |  |  |  |
| History of your respiratory diseases |  |  |  |  |  |  |  |
| History of your cardiac diseases |  |  |  |  |  |  |  |
| Body mass index (based on weight and height) |  |  |  |  |  |  |  |

End of Block: Covid-19 sensitivity

Start of Block: demographics

| 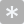 |
| --- |

In this last section, we are interested in some general information about yourself.

How old are you?

________________________________________________________________

| Page Break |  |
| --- | --- |

Please indicate your gender:

- Female
- Male
- Other

| Page Break |  |
| --- | --- |

Please indicate the size of the household you are currently living in:

- 1 person
- 2 persons
- 3 persons
- 4 persons
- more than 4 persons

| Page Break |  |
| --- | --- |

Please indicate your highest degree of education:

- Less than high school
- High school graduate
- Some college
- Bachelor's degree
- Master's degree
- Professional degree
- Doctorate

| Page Break |  |
| --- | --- |

Please select the U.S. state in which you are currently living in.

▼ Alabama ... Wyoming

| Page Break |  |
| --- | --- |

Do you own a smartphone?

- Yes
- No

| Page Break |  |
| --- | --- |

In general, would you say your health is?

|  | Poor | Fair | Good | Very good | Excellent |
| --- | --- | --- | --- | --- | --- |
| In general I would say my health is.... |  |  |  |  |  |

| Page Break |  |
| --- | --- |

Have you been tested for COVID-19?

- Yes
- No

| Page Break |  |
| --- | --- |

Have your family members or friends been tested for COVID-19?

- Yes
- No

| Page Break |  |
| --- | --- |

Have you been infected with COVID-19?

- Yes
- No
- Don't know

| Page Break |  |
| --- | --- |

Have your family members or friends been infected with COVID-19?

- Yes
- No
- Don't know

| Page Break |  |
| --- | --- |

How high do you perceive the risk of being infected by COVID-19 yourself?

|  | 1 | 2 | 3 | 4 | 5 | 6 | 7 |  |
| --- | --- | --- | --- | --- | --- | --- | --- | --- |
| Very low risk |  |  |  |  |  |  |  | Very high risk |

| Page Break |  |
| --- | --- |

How high do you perceive the risk that COVID-19 negatively affects your health?

|  | 1 | 2 | 3 | 4 | 5 | 6 | 7 |  |
| --- | --- | --- | --- | --- | --- | --- | --- | --- |
| Very low risk |  |  |  |  |  |  |  | Very high risk |

| Page Break |  |
| --- | --- |

Would you like to leave us a comment?

________________________________________________________________

| Page Break |  |
| --- | --- |

End of Block: demographics

Start of Block: End
